# Patient‐Derived Glioblastoma Organoids for AI‐Guided Prediction of Therapeutic Response

**DOI:** 10.1002/advs.78202

**Published:** 2026-11-09

**Authors:** Sandbhor Puja, Arun Bhavyadharshini, John Geofrey, Mudliyar Siddharth, Ghanekar Shubham, Moyiadi Aliasgar, Shetty Prakash, Singh Vikas, Sadwani Nidhisha, Garg Akshohini, Sahay Ayushi, Chatterjee Abhishek, Epari Sridhar, Gupta Tejpal, Dasgupta Archya, Goda Jayant

**Affiliations:** ^1^ Translational Nanomedicine and Bioengineering Lab Proton Therapy Centre Department of Radiation Oncology Clinical Research Centre (CRC) Advanced Centre for Treatment Research and Education in Cancer‐Tata Memorial Centre (ACTREC‐TMC) Kharghar Navi‐Mumbai Homi Bhabha National Institute Dr. E. Borges Marg Mumbai India; ^2^ Hasan Lab Advanced Centre for Treatment Research and Education in Cancer ‐Tata Memorial Centre (ACTREC‐TMC) Cancer Research Institute (CRI) Navi‐Mumbai India; ^3^ Future Industries Institute School of Pharmacy and Biomedical Science College of Health Adelaide University Adelaide South Australia; ^4^ Radiobiology Lab Department of Radiation Oncology Clinical Research Centre (CRC) and Homi Bhabha National Institute and Advanced Centre for Treatment Research and Education in Cancer‐ Tata Memorial Centre (ACTREC‐TMC) Dr. E. Borges Marg Mumbai India; ^5^ Department of Neurosurgery Tata Memorial Centre Homi Bhabha National Institute Dr. E. Borges Marg Mumbai India; ^6^ Department of Pathology Tata Memorial Centre Homi Bhabha National Institute Dr. E. Borges Marg Mumbai India

**Keywords:** artificial intelligence, glioblastoma patient‐derived organoids, precision oncology, tumor microenvironment

## Abstract

Glioblastoma (GBM) remains one of the most treatment‐refractory brain tumors, with poor survival driven by intratumoral heterogeneity, adaptive therapy resistance, and an immunosuppressive microenvironment that conventional preclinical models have failed to capture. Although these systems offer partial biological insights, none simultaneously preserve patient‐specific tumor architecture, microenvironmental complexity, and scalability needed for translational research. Patient‐derived GBM organoids address this gap by recapitulating 3D tumor architecture, glioma stem cell (GSC) hierarchies, extracellular matrix (ECM) interactions, and clinically relevant drug response phenotypes in a human‐specific system. This review examines GBM organoid biology and engineering, from GSC isolation and ECM scaffold design to maturation, vascular integration, and immune co‐culture. We discuss established platforms including NeoCOR and GLICO, and the rationale for incorporating functional vasculature, tumor‐immune microenvironment components, blood‐brain‐barrier (BBB), and blood‐cerebrospinal fluid barrier (BCSFB) interfaces. Critically, we propose that integrating artificial‐intelligence (AI) and machine learning (ML) with GBM organoid platforms represents a major opportunity in the field. Convolutional neural networks, ML‐based multi‐omics integration, drug‐genome correlation analysis, and predictive modelling of therapy resistance can transform organoid readouts into clinically actionable decisions. This AI‐organoid framework offers a path toward real‐time, patient‐specific therapy selection, supporting precision‐oncology workflows, informing clinical trial design, and reducing GBM therapy failure.

## Graphical abstract

Patient‐derived glioblastoma organoids provide clinically relevant models that preserve tumor heterogeneity and enable integrated AI‐driven analysis of imaging, molecular, and treatment‐response data. Their convergence with advanced analytics supports predictive, personalized therapeutic strategies and may accelerate translation from patient‐specific models to precision treatment decisions.

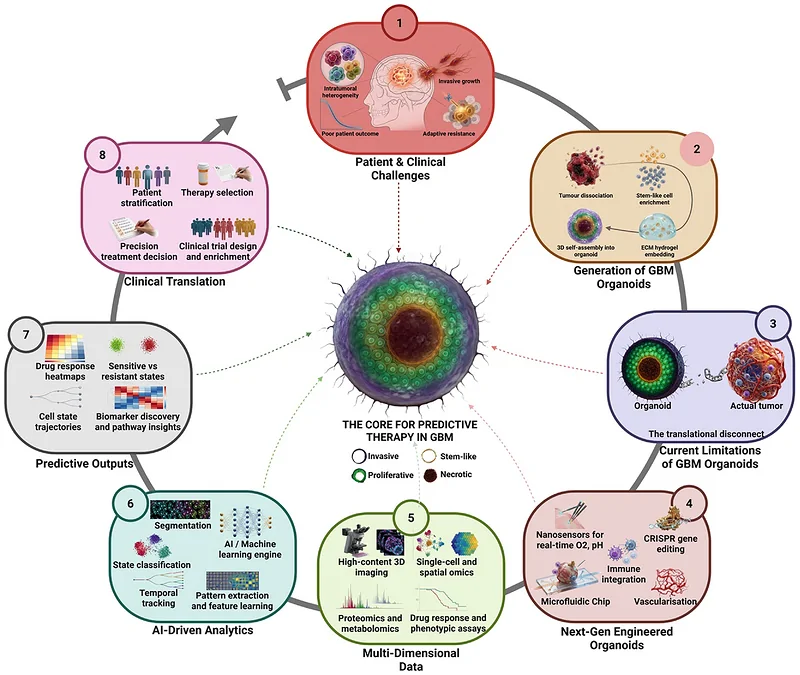

## 1 Introduction

Brain tumors, an intricate subset of neurological malignancies, contribute substantially to increasing mortality and morbidity across all age groups [1, 2, 3, 4]. Among primary brain tumors, gliomas are the most prominent, and glioblastoma (GBM) is the most common and aggressive brain tumor [5, 6]. According to the *Central Brain Tumor Registry of the United States* (CBTRUS), between 2017 and 2021, GBM accounted for 13.9% of all primary brain and other central nervous system tumors diagnosed in the United States [7]. Despite advancements in multimodal therapeutic approaches, the median overall survival (OS) of patients with GBM remains approximately 12–18 months [8], and the 5‐year survival rate is between 5% and 10% [9]. GBMs are characterized by extensive and abnormal angiogenesis, which hinders effective drug delivery to the tumor [10]. The tumor microenvironment (TME) exhibits significant cellular heterogeneity, including glioma stem cells (GSCs), found in perivascular niches (PVN) and the subventricular zones (SVZs), that drive tumorigenesis and recurrence [11, 12, 13, 14, 15]. Key genetic alterations in GBM include Epidermal Growth Factor Receptor (EGFR) amplification, Tumor Protein p53 (TP53) mutation, and loss‐of‐function in phosphatase and tensin homolog (PTEN), all contributing to its aggressive phenotype [16, 17]. Dysregulation of the retinoblastoma (RB) pathway further promotes uncontrolled proliferation, while Isocitrate Dehydrogenase (IDH) mutations, common in lower‐grade gliomas, are rare in GBM, highlighting distinct biological mechanisms. Risk factors for GBM include genetic predispositions along with environmental exposures such as ionizing radiation, viral infections, and obesity [18, 19]. Standard therapy involves maximal surgical resection followed by radiotherapy and chemotherapy with temozolomide (TMZ) [20]. Attempts to improve TMZ outcomes using O^6^‐benzylguanine (O^6^BG) have shown limited benefit, likely due to suboptimal dosing, poor *blood–brain barrier* (BBB) penetration, and variability in O^6^‐methylguanine DNA methyltransferase (MGMT) activity. Studies also highlight the importance of judicious treatment scheduling in clinical trials involving oncolytic virus therapies for GBM [21].

Novel therapeutic strategies include tumor‐treating fields (TTFields), which, when combined with TMZ, improve progression‐free survival (PFS) and OS but do not prevent recurrence [22]. Immunotherapies, such as immune checkpoint inhibitors (ICIs) and chimeric‐antigen receptor‐T cell (CAR T‐cell) therapy, show potential, although their efficacy is challenged by GBM's unique microenvironment [23, 24]. Recent candidates include machine learning (ML) ‐designed thioredoxin reductase inhibitors (4m and 4n), which reduced glioma progression in preclinical models without notable toxicity [25]. Furthermore, natural compounds such as rutin and quercetin enhance TMZ efficacy by improving bioavailability and BBB penetration [26, 27], while synthetic carboxamide derivatives such as BT‐851 and BT‐892 exhibit strong anti‐glioma activity in established GBM cell lines [28]. Despite these advancements, GBM remains highly lethal, emphasizing the need for more predictive and physiologically relevant experimental models. In this context, patient‐derived GBM organoids (PDGBOs) that preserve tumor heterogeneity and microenvironmental complexity provide a promising platform to better understand disease progression and treatment response. When complemented by advanced analytical approaches capable of interpreting complex biological data, these models can help bridge laboratory findings with clinically meaningful outcomes, thereby improving preclinical decision‐making and guiding more precise therapeutic strategies.

## 2 Existing GBM Models: A Brief Contextual Overview

Two‐dimensional (2D) cell culture models, where GBM cells grow as monolayers on flat substrates, have long served as the foundation for studying proliferation, migration, and invasion due to their simplicity, scalability, and compatibility with high‐throughput assays [29]. However, 2D systems fail to recapitulate the complex three‐dimensional (3D) TME, including extracellular matrix (ECM) architecture, oxygen and nutrient gradients, and interactions with stromal or immune components, frequently overestimating therapeutic efficacy compared to physiologically relevant systems [30]. Although co‐culture approaches partially improve microenvironmental representation, 2D cultures insufficiently model intratumoral heterogeneity, therapy‐induced resistance, and ECM‐regulated invasion central to GBM biology [31].

Patient‐derived xenograft (PDX) models preserve GBM heterogeneity [32], invasive behavior, and therapy response patterns, maintaining patient‐specific genomic, transcriptomic [33], and epigenetic landscapes [34, 35]. Orthotopic PDX models better recapitulate the TME and biological barriers, enabling evaluation of in vivo growth dynamics, BBB permeability, and systemic pharmacodynamics [36]. However, inconsistent engraftment, long generation times, high costs, and limited immune representation restrict their scalability and utility for high‐throughput or personalized testing [37, 38].

3D spheroid and tumoroid models provide an intermediate platform between 2D cultures and more complex in vivo systems, recapitulating hypoxia, nutrient gradients, and proliferative zonation observed in GBM [39]. While patient‐derived tumoroids retain higher genetic and phenotypic fidelity than cell line‐based spheroids and allow scalable drug screening, they often lack key components of the TME, including functional vasculature and BBB‐like properties [40, 41]. Although, microfluidic‐based 3D systems further introduce controlled gradients and perfusion, enhancing physiological relevance remain technically complex and limited in throughput [42, 43, 44]. Overall, existing GBM models, as represented in Table 1, each capture specific aspects of tumor biology but fail to simultaneously integrate patient‐specific heterogeneity, microenvironmental complexity, and experimental scalability, highlighting the need for advanced PDGBO‐ based platforms capable of bridging preclinical modelling and translational application.

**TABLE 1:** A comparative assessment of commonly used GBM models based on, architectural complexity, biological fidelity, scalability, cost‐efficiency, and translational relevance.

	2D Cell Culture	Spheroids and Tumoroids	Ex‐vivo Brain Slice Culture	Patient‐Derived Xenografts	Microfluidics (Organ‐on‐a‐Chip)
**Tumor Complexity and Architecture**	Monolayer; lacks 3D form; no polarity or gradients	**Spheroids**: Simple 3D aggregates; **Tumoroids**: Patient‐derived, retain heterogeneity and structural zones	Preserves full native architecture and anatomical integrity	Maintains tumor histology, especially in orthotopic models	Engineered microenvironments replicate tissue/tumor zones with compartmentalization
**Microenvironment Fidelity**	No ECM or stromal interaction	**Spheroids**: Limited stromal interaction; **Tumoroids**: Includes stromal, vascular, immune elements	Intact ECM, vasculature, and neural cells; native brain tissue preserved	Partial; orthotopic models better mimic TME than subcutaneous	Tailored ECM coatings, fluid shear stress, controlled stromal & vascular cues
**Immune System Incorporation**	Absent unless artificially co‐cultured	**Spheroids**: Immune simulation limited; **Tumoroids**: Can retain microglia and TILs	Preserves innate immunity (e.g., microglia); adaptive immune signals limited	Humanized mice enable partial immune relevance; immune suppression common	Human immune cells (e.g., T cells, macrophages) can be integrated for dynamic studies
**Scalability**	Highly scalable; ideal for screening	**Spheroids**: Moderate throughput; **Tumoroids**: Low scalability, patient variability	Poor scalability; tissue access and culture duration limited	Very low; variable tumor uptake and long latency	High‐throughput chips; multiplexing and automated readouts possible
**Time and Cost Efficiency**	Low cost; rapid turnaround (2–5 days)	**Spheroids**: Cost‐effective, quick; **Tumoroids**: Weeks to months, costly	Time‐consuming; lasts ∼1–2 weeks; expensive due to tissue prep	Expensive; takes months; requires animal housing and handling	Moderate‐to‐high initial setup; reusable platforms reduce long‐term cost
**Translational Relevance**	Low correlation with clinical outcomes	**Spheroids**: Partial mimicry; **Tumoroids**: High patient relevance, suitable for precision oncology	High local fidelity; lacks systemic context	High for chemotherapy; immunotherapy limited without humanization	Human‐relevant, dynamic drug response; mimics BBB, flow, immune‐tumor interface; promising for precision trials

* TILs‐ Tumor Infiltrating Lymphocytes, GEMMs‐ Genetically engineered mouse models

## 3 Bridging the Gap: Challenges in Translational GBM Research

Translational GBM research is hindered by the lack of a universal model that captures patient heterogeneity. Preclinical platforms often require complex surgeries, advanced imaging, or large animal cohorts, constrained by variability, surgical risks, and ethical considerations, slowing clinical translation [45]. Replicating the complex TME, including 3D architecture, vascular integration, and immune dynamics remains a major challenge. Even advanced in vitro systems struggle to mimic tumor–immune interactions and hypoxic gradients [46]. Intratumoral heterogeneity‐driven by genetic, epigenetic, and metabolic changes‐further complicates translation, promoting therapy resistance and recurrence [47]. Models that do not reflect this diversity tend to have limited predictive value, which partly explains why so many GBM therapies fail in clinical trials [48, 49]. The immune system plays a critical role in GBM progression but remains underrepresented in current models. Immunotherapies successful in other cancers show limited efficacy in GBM due to this translational gap. While PDXs preserve tumor histology and molecular traits, they lack a functional immune system, restricting immunotherapy studies. Advances in humanized mouse models incorporating human immune cells show promise, but the immunosuppressive GBM TME, shaped by tumor‐associated macrophages (TAMs), monocyte‐derived macrophages (MDMs), and regulatory T‐cells (Treg), still hinders effective therapy design [50]. Another significant challenge in GBM treatment is the presence of both the BBB, blood‐cerebrospinal fluid barrier (BCSFB) and blood‐brain‐tumor barrier (BBTB), which restricts the penetration of systemically administered drugs. Unlike the relatively uniform BBB, the BBTB shows heterogeneous permeability. Strategies to overcome this include nanoparticles with tumor‐targeting ligands, engineered extracellular vesicles for selective BBB transcytosis [51], and focused ultrasound (FUS) therapy combined with microbubble cavitation to transiently disrupt the BBTB [52, 53]. While promising, these approaches are still experimental, requiring further optimization for safe and effective clinical translation.

Single‐cell sequencing enables high‐resolution analysis of GBM heterogeneity, identifying therapy‐resistant subpopulations and mapping tumor evolutionary trajectories [54]. Integrating multi‐omics (metabolomics, proteomics) further clarifies molecular drivers of progression. Yet, despite these advances, reliable biomarkers for patient stratification remain difficult to identify, largely because of GBM's ability to adapt and develop resistance [49]. Key candidate biomarkers in GBM include MGMT methylation, predicting TMZ response [55], EGFRvIII mutations, associated with aggressive growth and therapeutic targeting [56], and IDH1/2 mutations, associated with better prognosis. Emerging markers like Telomerase Reverse Transcriptase (TERT) promoter mutations and Programmed Death‐Ligand 1 (PD‐L1) expression are under investigation for their roles in tumor progression and immunotherapy response [57]. Interestingly, imaging biomarkers, including radiomic MRI features, have been shown to noninvasively stratify GBM patients by risk and treatment response, complementing molecular markers for improved clinical trial design [58, 59]. Translating preclinical findings into effective GBM therapies requires innovative trial designs that account for tumor molecular diversity. Adaptive approaches, such as basket and umbrella trials, enable efficient evaluation of targeted therapies in stratified cohorts, though patient recruitment, ethics, and regulatory hurdles remain significant [60]. Bridging this translational gap demands a multidisciplinary strategy integrating tumor biology, advanced imaging, computational modelling, and PDGBO platforms to accelerate effective GBM treatment development [61, 62].

## 4 Patient‐derived GBM Organoids: A Revolutionary Tool

Given the limitations of existing GBM models, PDGBOs offer a promising platform for studying tumor progression and therapy response. These 3D, self‐organizing structures derived from induced pluripotent stem cells (iPSCs) or tumor tissue replicate organ architecture and function [63], providing a more accurate representation of the TME than 2D cultures or cell‐line xenografts [64]. By preserving cellular heterogeneity, stromal interactions, and dynamic microenvironmental changes, organoids are valuable for personalized medicine and biomarker discovery [49]. Their ability to recapitulate 3D tumor architecture allows more precise modelling of tumor behavior and treatment outcomes. PDGBOs can incorporate key features difficult to model in 2D cultures or animals, including immune cells, BBB components, and single‐cell genetic diversity [65]. For instance, Hubert et al. in 2016 generated PDGBOs preserving genetic and phenotypic heterogeneity, including GSCs and hypoxic gradients, enabling studies of tumor evolution and treatment resistance [66]. Complementing this, in 2020 Jacob et al. leveraged PDGBOs for high‐throughput drug screening, assessing sensitivity to targeted therapies and immunotherapies to identify patient‐specific treatment regimens [67]. Single‐cell RNA sequencing enables mapping of cellular heterogeneity and identification of recurrence‐driving populations, while CRISPR‐based approaches allow functional interrogation of oncogenic mutations [68]. Vascularized organoids further enable modelling of angiogenesis and testing of anti‐angiogenic therapies [69]. PDGBOs also provide a platform to study immune‐tumor interactions. By integrating immune cells, BBB components, and patient‐specific genetic variations, organoids mimic the GBM microenvironment more accurately than 2D cultures or animal models [70]. Unlike 2D systems, organoids enable immune cells to interact with GBM cells in a 3D spatial context, capturing processes such as PD‐L1–mediated immune evasion and cytokine signaling [71, 72]. Incorporation of endothelial cells and astrocytes enables evaluation of drug penetration, immune trafficking, and barrier‐associated effects on therapy delivery. By preserving mutations such as IDH1/2, EGFRvIII, and TERT promoter alterations, organoids reflect intratumoral heterogeneity, clonal evolution, and therapy resistance [73]. Innovations including vascularized organoids and microfluidic integration further enable modelling of angiogenesis, perfusion, and shear stress, supporting functional evaluation of immunotherapies such as CAR‐T cells, ICIs, and oncolytic viruses [74, 75]. While long‐term culture stability remains a practical hurdle, PDGBOs offer something conventional models have long fallen short on: a human‐specific system that more faithfully captures the biology and therapeutic complexity of the disease.

## 5 Development of PDGBOs

### 5.1 Sourcing Glioblastoma Stem Cells

The establishment of PDGBOs relies on the isolation of GSCs, which preserve the intrinsic heterogeneity and aggressive features of patient tumors [76, 77]. These cells are central to modelling GBM biology, as they drive proliferation, invasion, and therapy resistance. GSC enrichment is commonly achieved using complementary strategies including fluorescence‐activated cell sorting (FACS), magnetic‐activated cell sorting (MACS), sphere‐formation assays, and immunohistochemistry, with selection based on stem cell‐associated markers such as CD133 and CD44 [78, 79, 80]. Functional validation through sphere formation, differentiation capacity, and tumorigenicity assays in immunocompromised mice confirms stem‐like properties and relevance to clinical aggressiveness [81, 82].

Freshly resected tumor tissue remains the most common source for GSC isolation, enabling sampling from both tumor cores and invasive margins [83]. Core regions are enriched in proliferative, hypoxia‐adapted GSCs, whereas invasive margins harbor quiescent, therapy‐resistant, and highly migratory populations [66]. Inclusion of cells from both compartments improves representation of spatial heterogeneity, invasion dynamics, and therapeutic response. Organoids have also been generated directly from minimally dissociated tumor fragments, preserving native tissue architecture and tumor microenvironmental features [84]. In parallel, core needle biopsies provide a minimally invasive source for patient‐specific organoid generation, expanding clinical applicability [85]. Advanced genetic engineering approaches further extend the utility of GSC‐ and tissue‐derived organoids. Integration with CRISPR/Cas9‐based editing enables modelling of patient‐specific driver alterations, while iPSC‐derived cerebral organoids harboring defined mutations (e.g., IDH, EGFR) allow systematic interrogation of mutation‐dependent tumor behavior, drug response, and resistance mechanisms [68, 86, 87, 88, 89]. Together, these strategies ensure that organoids retain biological fidelity and translational relevance.

### 5.2 Constructing the Extracellular Matrix

Reconstruction of a physiologically relevant ECM is essential for supporting GSC survival, self‐renewal, invasion, and therapeutic response within PDGBOs [90], since the ECM provides both structural support and biochemical/mechanical cues that govern tumor–stromal behavior [91]. A comparison of commonly used ECM types is provided in Table 2. Matrigel – enriched in laminin, collagen IV, and entactin, remains the most widely used substrate for supporting GSC maintenance and invasive growth [92]. Matrix stiffness, driven largely by collagen‐I content, is itself a key regulator of proliferation, invasion, and therapy resistance, with GBM tissue substantially stiffer than normal brain [93]; this can be tuned via collagen concentration or synthetic additives such as polyethylene glycol (PEG) [94, 95]. Hyaluronic acid, a major brain‐ECM component, adds CD44‐mediated invasion and stemness signaling [96], while fibrin, fibronectin, and laminin further contribute integrin‐mediated adhesion, migration, and angiogenic cues [97]. Hypoxia, a hallmark of GBM, is reproduced through ECM‐generated oxygen gradients or low‐oxygen culture, activating HIF pathways and enriching GSC populations [98, 99]. Collectively, these ECM strategies let PDGBOs integrate the biochemical, mechanical, and metabolic signals that define the in vivo tumor microenvironment.

**TABLE 2:** Comparison of ECM Types for GSC Culture and GBM Organoid Modeling.

Sr No.	ECM Type	Supports GSC Self‐Renewal and Tumorigenicity	Influence on Organoid Behavior	Advantages	Limitations	References
1.	Matrigel	Strongly supports GSC maintenance and sphere formation	Promotes invasive growth and pseudo‐stratified tumor architecture	Rich in laminin, collagen IV, and growth factors; well‐established	Batch‐to‐batch variability; animal‐derived; lacks tunability	[100]
2.	Collagen I	Supports adhesion and moderate GSC proliferation	Stiffer gels promote invasion; modulates mechanosensing	Defined structure; tunable stiffness; more consistent than Matrigel	Poor GSC self‐renewal when used alone; lacks GBM‐specific ECM cues	[101]
3.	Fibrin	Supports 3D invasion and migration of GSCs	Promotes angiogenesis‐like behavior in co‐culture models	Biocompatible; involved in wound healing; dynamic remodeling	Short gel lifetime; not GBM‐specific; requires co‐factors	[102]
4.	Hyaluronic Acid (HA)	Highly relevant for GBM due to abundance in brain ECM	Promotes invasion and CD44‐mediated signaling; enhances GBM aggressiveness	Brain‐specific; modulates cell motility and stemness	Often used in combination; requires chemical modification for stiffness	[103]
5.	PEG‐based Synthetic ECMs	Supports tunable GSC culture when bioactive motifs (e.g., RGD, HA) are included	Highly controllable microenvironment; allows modelling of stiffness‐ or ligand‐specific effects	chemically defined; reproducible; allows precise biophysical tuning	Requires functionalization to mimic native ECM; may lack natural cues	[104]

### 5.3 Manufacturing, Maturation, and Storage Considerations

Scalable generation of PDGBOs requires culture conditions that preserve stemness, heterogeneity, and tumor‐defining features. Growth factor supplementation, particularly EGF and FGF2, supports GSC proliferation and maintenance of malignant phenotypes [105]. Bioreactor‐based systems and dynamic culture conditions improve nutrient and oxygen exchange, reduce necrotic core formation, and support the generation of larger, more uniform organoids, while maintaining intratumoral diversity. Co‐culture with non‐malignant brain cells including astrocytes, neurons, microglia, oligodendrocyte precursor cells, and endothelial cells further enhances microenvironmental complexity and biological relevance [106, 107].

As organoids mature, intrinsic developmental processes and ECM composition shape structural organization, hypoxic gradients, and cellular heterogeneity [108]. Natural matrices such as Matrigel and collagen‐I differentially influence GSC maintenance, invasion, and mechano‐transduction pathways, while fibrin and hyaluronic acid better replicate perivascular and angiogenic niches. Synthetic ECMs offer better reproducibility and tunability, but at the cost of full biological complexity a trade‐off that remains a genuine challenge when physiological fidelity is the goal to improve prediction precision [109].

Long‐term utility of PDGBOs depends on effective cryopreservation strategies that maintain cellular heterogeneity and patient‐specific genomic features [110]. Both slow freezing and vitrification approaches have been used successfully, provided cryoprotectant exposure and cooling rates are carefully optimized to minimize cellular damage and population loss [111]. The establishment of GBM organoid biobanks enables long‐term storage, sharing, and reuse of PDGBOs, supporting personalized therapy testing and large‐scale drug screening efforts [112]. Collectively, manufacturing, maturation, and storage considerations are integral to maintaining the translational value of PDGBOs rather than serving as purely technical steps. Moreover, Figure 1 outlines the stepwise workflow for generating PDGBOs, from tumor tissue acquisition to cryopreservation, illustrating how each stage contributes to biological fidelity and translational relevance.

**FIGURE 1:**
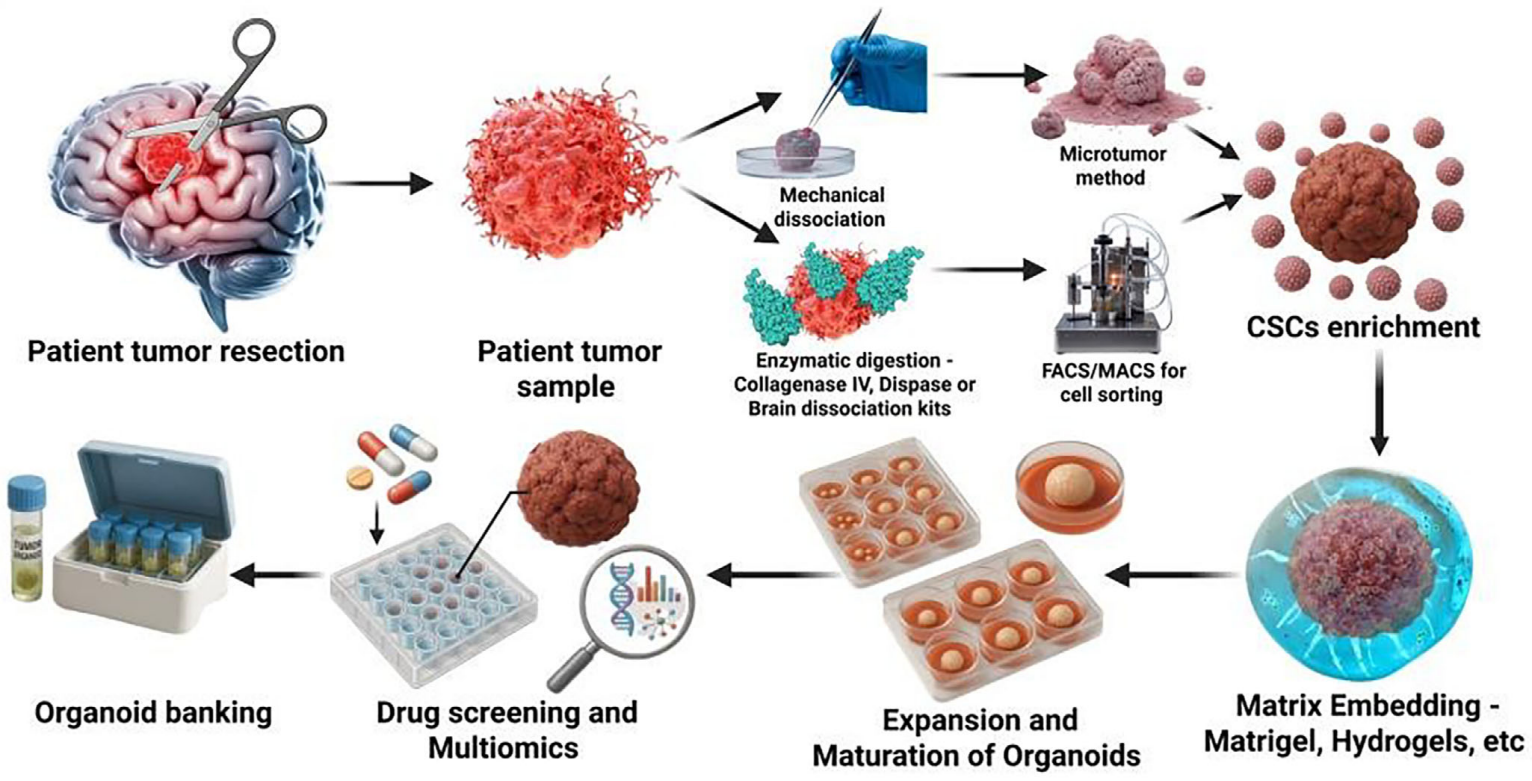
Schematic representation of the stepwise workflow generating patient‐derived GBM Organoids.

## 6 Available GBM Organoid Models

As discussed previously, given the biological complexity and clinical heterogeneity of GBM, no single 3D model adequately captures all aspects of tumor initiation, progression, and therapeutic response. Instead, GBM organoid platforms differ in genetic controllability, microenvironmental fidelity, scalability, and translational relevance. To support rational experimental design, Table 3 summarizes the alignment between specific research questions and the most appropriate GBM 3D models. The subsections below discuss representative platforms with emphasis on their functional use cases rather than descriptive classification.

**TABLE 3:** Model selection guide for GBM 3D platforms.

Research Question / Experimental Goal	Recommended Model	Rationale	References
Rapid drug sensitivity testing using patient material	Tumoroids / Patient‐derived GBM Organoids (PDGBOs)	Preserve patient‐specific genetic and phenotypic features; scalable for parallel drug screening	[113]
Studying early gliomagenesis and genotype–phenotype relationships	NeoCOR	Enables controlled introduction of defined oncogenic mutations within developing neural tissue	[68]
Dissecting tumor–brain developmental interactions	NeoCOR	Contains neoplastic and non‐neoplastic zones, allowing direct observation of tumor–brain interface	[68]
Investigating GBM invasion, migration, and tissue infiltration	GLICO	Recapitulates GBM cell infiltration into human cerebral organoid cytoarchitecture	[114]
Modeling tumor heterogeneity and variable therapy response	GLICO / Patient‐derived PDGBOs	Maintains inter‐ and intra‐tumoral heterogeneity seen in patient tumors	[48]
Precision oncology and personalized therapeutic testing	Patient‐matched GLICO or PDGBOs	High translational fidelity when derived from patient‐specific tumor material	[115]
Mechanistic studies with defined genetic background	NeoCOR	Genetic manipulation is programmable and reproducible	[68]
High‐throughput mechanistic assays with limited complexity	Spheroids	Simple, scalable, cost‐effective but limited microenvironmental relevance	[116, 117]
Studying ECM dependence and hypoxia‐driven resistance	Organoids / Tumoroids	Maintain 3D gradients and ECM interactions absent in 2D systems	[116]
Evaluating angiogenesis, immune modulation, or BBB penetration	Next‐generation integrated GBM organoids	Incorporate vascular, immune, and microfluidic components	[117]
Longitudinal evolution and therapy adaptation	PDGBOs	Allow expansion, cryopreservation, and repeated testing over time	[118]

* NeoCOR: neoplastic cerebral organoid; GLICO: glioblastoma–cerebral organoid

### 6.1 NeoCOR Model: Genotype‐Driven Gliomagenesis and Tumor–Brain Interactions

The neoplastic cerebral organoid (NeoCOR) model was developed to overcome limitations in studying early gliomagenesis by enabling controlled tumor initiation within a human neural developmental context [68]. In this system, oncogenic transformation is induced during cerebral organoid formation through targeted genetic manipulation using CRISPR–Cas9 or transposon‐based approaches. Activation of oncogenes such as MYC, together with loss of tumor suppressors including TP53 and NF1, results in neoplastic outgrowths embedded within otherwise non‐transformed brain‐like tissue. This architecture permits direct interrogation of tumor emergence, invasion, and interaction with surrounding neural compartments. NeoCOR‐derived tumors exhibit transcriptomic and histopathological similarity to patient GBM and retain tumorigenic potential upon in vivo transplantation. Importantly, the ability to introduce defined genetic alterations enables causal dissection of genotype‐phenotype relationships that are difficult to resolve in patient‐derived models, where heterogeneity is inherited rather than experimentally controlled. NeoCOR is therefore best suited for studies of glioma initiation, mutation‐specific biology, and tumor–brain developmental interactions. Despite these advantages, NeoCOR primarily models genetically driven tumor initiation rather than patient‐specific tumor evolution. The absence of vascular, immune, and BBB components limits its capacity to capture angiogenesis, immune modulation, and therapeutic delivery constraints [88].

### 6.2 GLICO Model: Tumor Invasion and Patient‐Specific Microenvironmental Interactions

For investigations focused on GBM invasion, migration, and interaction with human brain cytoarchitecture, the glioblastoma–cerebral organoid (GLICO) model represents a preferred platform [118]. Initial work in 2018 by da Silva et al. demonstrated that GSC‐derived spheroids can successfully attach to and infiltrate immature cerebral organoids, laying the foundation for this model [119]. Linkous et al. further refined it using human embryonic stem cell–derived cerebral organoids co‐cultured with patient‐derived GBM cells [114]. GLICO holds a distinct advantage because it co‐cultures patient‐derived GSCs with human cerebral organoids (Brain Organoid‐Brain Tumor or BO‐BT model) which helps in creating native cytoarchitecture, ECM and cellular diversity of the brain which conventional spheroid or monocultures lack. Importantly, GLICO organoids replicate intertumoral heterogeneity in treatment response, displaying variable sensitivities to chemotherapy and radiotherapy [120, 121].

Unlike NeoCOR, which induces transformation in situ, GLICO introduces patient‐derived GSCs into pre‐formed cerebral organoids, allowing established tumor cells to interact with an organized human neural microenvironment. Within this system, GSCs infiltrate cerebral organoids and recapitulate invasive growth patterns characteristic of patient tumors. The presence of structured neural regions, enables assessment of spatially defined invasion and migration behaviors that are not captured by spheroid or monoculture models. When generated using patient‐matched tumor and cerebral tissues, GLICO organoids preserve interpatient heterogeneity and exhibit variable responses to therapeutic agents, supporting their application in functional precision oncology. However, similar to NeoCOR, current GLICO platforms lack functional vasculature and immune compartments, leaving immune surveillance, neuroinflammatory signaling, and drug penetration across the BBB incompletely modelled [108].

Collectively, the complementary strengths and limitations of NeoCOR and GLICO highlight that no single current platform can simultaneously recapitulate patient‐specific tumor heterogeneity, defined genetic control, brain architecture, and the vascular and immune components of the GBM microenvironment. These gaps have prompted the development of next‐generation integrated PDGBOs that combine patient‐derived tumor biology with engineered microenvironmental components to improve physiological fidelity and translational relevance.

### 6.3 Next‐Generation Integrated PDGBOs: Toward Inclusive Tumor–Brain System Modelling

These next‐generation systems incorporate bioengineered ECM scaffolds, microfluidic perfusion, and vascular‐like networks to better model nutrient gradients, hypoxia, angiogenesis, and therapeutic delivery. Parallel efforts focus on introducing immune components, including microglia and peripheral immune cells, to enable investigation of tumor‐immune interactions and immunotherapy response. Such hybrid platforms represent a critical step toward recapitulating the dynamic and multicellular GBM ecosystem while retaining experimental control and reproducibility. Unlike earlier descriptive models, these systems integrate genetic, microenvironmental, and therapeutic dimensions, making them better suited to address questions that actually matter in the clinic.

Compared with spheroids, organoid‐based systems more accurately reproduce 3D architecture, hypoxic gradients, and intercellular signaling [122, 123], while PDGBOs specifically preserve native tumor histology and molecular heterogeneity for therapeutic screening and biomarker discovery [124]. Realizing this advantage in practice still requires resolving formation variability, limited scalability, and a lack of standardized quality control, alongside continued incorporation of vascular and immune components [125]. Ultimately, GBM organoid models should be selected based on the specific biological question and required microenvironmental complexity, rather than model nomenclature alone.

## 7 Limitations of GBM Organoid Models and Their Experimental Implications

GBM organoid platforms advance meaningfully beyond 2D cultures and spheroids by capturing 3D architecture, clonal heterogeneity, and stem‐like compartments, yet remain reductionist representations of GBM biology. Their limitations are not merely technical, they define the scope of biological processes that can be reliably studied, with direct consequences for experimental design, data interpretation, and translational integration. The following sections examine these limitations alongside potential solutions and their implications for organoid modelling.

### 7.1 Lack of Functional Vasculature and Its Impact on Tumor Modelling

A defining limitation of most GBM organoid platforms is the absence of functional vascular networks: neuroectoderm‐biased differentiation protocols and diffusion‐limited culture conditions restrict the formation of endothelial cells, pericytes, and perfusable microvessels [126, 127], so oxygen, nutrient, and drug transport relies on passive diffusion, producing artificial hypoxia and necrosis in larger constructs [69, 128]. This avascularity broadly limits representation of angiogenesis, endothelial‐tumor signaling, and BBB‐mediated drug delivery [129, 130, 131, 132], and precludes evaluation of anti‐angiogenic therapies, vascular normalization strategies, and vascular‐remodeling‐driven resistance [133, 134]. Consequently, therapeutic responses observed in avascular organoids may reflect nutrient stress‐induced cytotoxicity rather than clinically relevant pharmacodynamic effects [135, 136, 137, 138] – a caveat essential to interpreting organoid‐based drug response data.

The hyper vascular phenotype of GBM and its capacity for adaptive vascular remodeling remain major obstacles to durable therapy [139], and the modest benefit of VEGF‐targeted agents shows that GBM neovascularization is not one pathway but a composite, mutually compensating program of angiogenesis, vasculogenesis, vascular mimicry, endothelial transdifferentiation, and vessel co‐option [140]. Angiogenesis, the best‐characterized route, proceeds through HIF‐1α–driven VEGF induction and MMP‐mediated vessel remodeling [141], because avascular PDGBOs lack endothelial and mural cells, they cannot model vessel sprouting, maturation, or therapy‐induced vascular normalization/collapse [142, 143, 144]. Vasculogenesis, driven by endothelial progenitor and TAM recruitment via SDF‐1/ C‐X‐C Motif Chemokine Receptor 4 (CXCR4) signaling after radiotherapy or anti‐angiogenic treatment [145], is similarly unattainable without circulating progenitor pools or stromal recruitment. Vascular mimicry, endothelial‐free perfusable channels linked to mesenchymal, stem‐like phenotypes and radioresistance [146, 147], cannot be reproduced without flow, shear stress, or dynamic ECM remodeling. GSC‐derived tumor endothelial cells (TDECs) retaining oncogenic alterations such as EGFR amplification arise via HIF‐1α–driven lineage conversion, amplified by radiotherapy, TMZ, or VEGF blockade [148, 149, 150, 151]. PDGBOs lack the endothelial differentiation cues and perfusion needed to evaluate this [152, 153, 154]. Vessel co‐option – glioma cells hijacking pre‐existing vasculature at invasive margins via Cdc42/CD44‐mediated pericyte engagement [155, 156, 157, 158], is likewise unmodellable in avascular systems. Together these redundant, compensatory mechanisms [159, 160] explain why single‐pathway antiangiogenic therapies fail, and why none is currently captured by avascular organoid platforms; addressing this will require vascularized, perfused organoid‐on‐chip systems.

Rather than a single “fully vascularized” construct, a stratified design logic, matching specific neovascular mechanisms to defined experimental questions – will better serve biological fidelity and translational relevance. Figure 2 illustrates how vascular, immune, stromal, and microfluidic integration in next‐generation platforms addresses the limitations discussed above.

**FIGURE 2:**
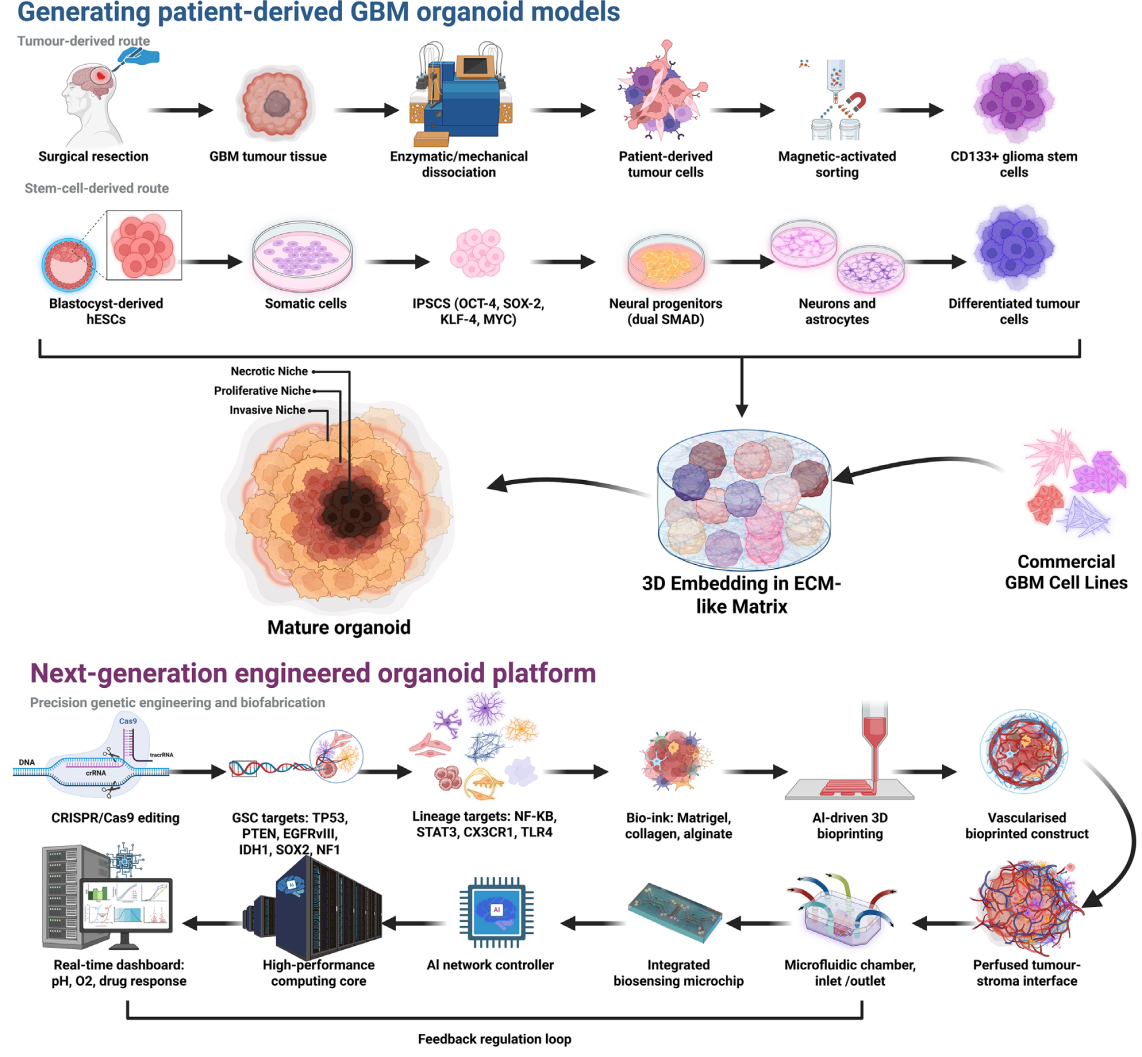
Comparative schematic of current and next‐generation GBM organoid models. (A) Current organoid systems are derived from patient tumor samples, glioma stem‐like cells (GSCs), differentiated tumor cells, or stem cell‐derived neural lineages. These cells are embedded in ECM‐based hydrogels such as Matrigel to form 3D structures. While these models offer partial representation of tumor biology, they are limited by the absence of vascular networks, immune components, and stromal cells. This results in restricted nutrient and oxygen diffusion, central necrosis, poor drug penetration, and a lack of real‐time monitoring capabilities. Additionally, they often fail to capture the cellular and spatial heterogeneity of in vivo GBM. (B) The future GBM organoid model integrates gene‐edited cells using CRISPR technology to mimic key tumor mutations and cellular behaviors. These cells, including immune, neural, vascular, and tumor types, are spatially arranged using AI‐assisted 3D bioprinting onto defined scaffolds. The printed constructs are maintained within a microfluidic culture system that continuously supplies nutrients, oxygen, and therapeutic agents. Embedded sensors monitor the internal environment including pH, oxygen levels, and drug effects in real time. Data from these sensors are processed by a central computing system with artificial intelligence, enabling automated control of environmental parameters and predictive modelling of tumor behavior. This integrated platform more closely simulates the human TME and supports dynamic testing of therapies in a scalable, reproducible format. A related, less‐studied barrier is the BCSFB formed primarily by the choroid plexus epithelium, which regulates CSF composition and blood‐CNS trafficking via specialized tight junctions and transport systems [161, 162, 163]. In vitro BCSFB models — polarized epithelial monolayers, co‐cultures, and CSF‐mimetic organoids [164] – offer a route to study intrathecal delivery, perivascular niche interactions, immune trafficking, and CSF‐mediated dissemination if integrated with PDGBOs [165].

Endothelial cells and astrocytes further shape GBM progression through direct signaling with tumor cells – chemokines, cytokines, growth factors, extracellular vesicles, and gap junctions [166, 167, 168, 169] – regulating GSC proliferation, self‐renewal, and stemness [184, 185, 186, 187]. Reactive astrocytes secrete Connective Tissue Growth Factor (CTGF), IL‐6, and Stromal Cell‐Derived Factor 1 (SDF‐1) via Elevated Gene‐1 (AEG‐1) – dependent signaling to enhance invasion [170, 171], while endothelin / Protein Kinase B / Mitogen‐Activated Protein Kinase (AKT/MAPK) signaling and astrocyte‐derived glutamine drive chemoresistance and nutrient support under stress [172, 173]. ECs reinforce GSC niches through endothelial‐derived nitric oxide (NO), Notch ligands, and Sonic hedgehog (Shh), sustaining stem‐like programs and survival [172], and reciprocal tumor–stroma crosstalk remodels ECM composition and stiffness [174, 175] – laminin‐α2 upregulation limits drug penetration, while proteases (uPA, cathepsins, MMP9) drive endothelial migration and pro‐angiogenic factor release [174, 175, 176]. None of this endothelial–tumor crosstalk, perivascular niche maintenance, or ECM‐vascular feedback is captured by non‐vascularized PDGBOs, which model only tumor‐intrinsic stemness and hypoxia‐responsive transcription. GSC behavior is further organized by spatially distinct vascular niches – perivascular, periarteriolar, necrotic, and invasive – each imposing distinct biochemical and metabolic signals on stemness, dormancy, and resistance, enabling stem/differentiated interconversion after therapy [77, 177, 178].

The PVN, comprising ECs, astrocytes, perivascular stromal cells (PSCs), and GSCs [179], sustains stemness through Notch/Shh/NO signaling while GSCs reciprocally drive angiogenesis via VEGF and transdifferentiation [180, 181, 182]; proximity to vessels confers survival advantage, while distal quiescent cells resist therapy [183, 184, 185, 186, 187], and PSC‐secreted factors reinforce invasion, dormancy, and TMZ resistance [181]. This niche also imposes metabolic zonation, with vessel‐proximal cells favoring mTOR‐driven oxidative phosphorylation over glycolysis [188].

Within the periarteriolar niche (PAN) – first described by Hira et al. (2015) – hypoxia sustains CD133+/Nestin+ GSC populations adjacent to CD31+ endothelium via CXCR4/CD44/SDF‐1α/OPN signaling [189, 190]; the SDF‐1α/CXCR4 axis anchors GSCs within PANs, whereas the protease Cathepsin K (CTSK) degrades SDF‐1α, releasing CXCR4^+^ GSCs for migration and invasion. This CTSK–SDF‐1α balance regulates GSC localization within hypoxic gradients [221]. Therapeutically, CXCR4 is overexpressed in over 50% of GBMs, especially in pseudo‐palisading and microvascular regions. Gravina et al. (2017) showed that the CXCR4 antagonist PRX177561 reduced GSC proliferation, migration and EMT markers, promoted M1 macrophage polarization and suppressed tumor vasculature. Combined with bevacizumab or sunitinib, PRX177561 delayed tumor progression and improved survival in preclinical models, highlighting the translational relevance of targeting PAN‐specific pathways [191]. More severe hypoxia in the necrotic niche selects for pseudo‐palisading, therapy‐resistant GSCs [192], sustained by hypoxia‐induced lncRNAs and ABC transporters [193], and HIF‐1α/2α–driven stemness and invasion programs (TGF‐β, Notch, VEGF, JAK/STAT, EMT regulators) [194, 195, 196, 197, 198, 199].

At the invasive margin, GSCs infiltrate via vessel co‐option and white‐matter migration [200], driven by MMP‐2/9–mediated ECM remodeling, FABP6, and hypoxia/anti‐VEGF–induced MET–EMT programs [99, 201, 202], and reinforced by astrocyte/microglia signaling (CTGF–NF‐κB–ZEB1, SHH–GLI, EGF) that also suppresses macrophage phagocytosis [171, 203, 204]. For instance, microglia‐derived EGF enhances glioma migration, while GSC‐derived signals suppress macrophage phagocytosis and induce IL‐10 and TGF‐β1, creating an immunosuppressive environment [205]. Additionally, glioma cells engage in perineuronal satellitosis, clustering around neurons. Neuron‐derived neuroligin‐3 activates PI3K–mTOR signaling, creating a feed‐forward proliferative loop. However, current avascular PDGBOs fail to recapitulate these vascular, immune, and neuroglial interactions, limiting faithful modelling of invasion and therapeutic resistance.

Collectively, none of these niche‐specific oxygen gradients, metabolic zonation, vascular co‐option dynamics, or endothelial–stromal crosstalk mechanisms are captured by current avascular PDGBOs, where hypoxia arises from diffusion limits rather than genuine vascular pathology. Faithful modelling will require vascularized, perfused, multicellular platforms integrating endothelial, stromal, immune, and neural components.

### 7.2 Lack of Tumor Immune Microenvironment (TIME)

Although the CNS was historically considered immune‐privileged, it retains innate and adaptive immune capacity – immune cells traffic to perivascular spaces, antigens reach cervical lymph nodes via Virchow‐Robin spaces, and immunoglobulins cross the BBB via FcRn‐mediated transport, with BBB integrity further compromised during inflammation [206].

TAMs are the dominant immunoregulatory component of GBM. Recruited via C‐C Motif Chemokine Ligand 2 (CCL2), C‐X‐C Motif Chemokine Ligand 12 (CXCL12), and Colony Stimulating Factor 1 (CSF‐1), and enriched in hypoxic/invasive regions, M2‐polarized TAMs secrete TGF‐β, IL‐10, VEGF, and MMPs, sustaining GSC maintenance, ECM remodeling, and immune evasion through STAT3/TGF‐β signaling [207, 208, 209]; current PDGBOs, lacking myeloid compartments, cannot model this axis.

TAMs actively drive GBM invasion and therapeutic resistance through coordinated cytokine, protease, and innate immune signaling [209, 210, 211, 212]. PDGBOs lacking TAMs cannot model this macrophage‐dependent invasion.

TAMs are equally central to therapy resistance: exosomal miR‐21 transfer activates STAT3/TGF‐β stemness pathways to confer TMZ resistance, and radiotherapy reshapes TAM phenotype to support recurrence [213, 214, 215], while PD‐L1^+^ TAMs limit checkpoint efficacy, though combinatorial targeting of PI3Kγ, CSF‐1R, or STAT5 restores sensitivity preclinically [216, 217].

Lymphoid dysfunction compounds this suppression: infiltrating CD8^+^/CD4^+^ T cells become functionally exhausted via checkpoint expression, TGF‐β/IL‐10 signaling, PD‐1–PD‐L1 interactions, and MDSC‐mediated suppression [218, 219, 220], while regulatory T cells are preferentially recruited via CCL22–CCR4/IDO pathways and correlate with poor survival [221, 222]. Without functional lymphoid compartments or antigen presentation, PDGBOs cannot model checkpoint resistance, Treg‐mediated suppression, or patient‐specific immunotherapy response [223, 224].

### 7.3 Control on Spatial Dynamics

In GBM, the paracrine and direct intercellular communication landscape is highly dependent on spatial context, and this can vary substantially across different organoid and 3D model systems where the microenvironmental distances between cells influence signaling gradients and functional outcomes. Tunneling nanotubes (TNTs) ‐ long membranous channels formed between GBM cells, have been implicated in long distance intercellular transfer of organelles such as mitochondria and adaptive metabolic signals, contributing to microenvironment adaptation and potentially therapy resistance in spatially heterogeneous tumor niches [225, 226]. In parallel, gap junction mediated communication via connexin proteins, including Cx43, enables exchange of small signaling molecules and ions between adjacent cells and has been shown to regulate invasion, stem like maintenance, and phenotypic plasticity in GBM and GSCs [227, 228]. Together, these modes of contact dependent communication mediated by TNT networks and gap junction channels emphasize the importance of studying spatially resolved communication mechanisms in organoid models, as compactness and cell‐cell distances can drastically alter the balance and functional impact of these pathways.

## 8 Opportunities in Clinical Modelling Implications

Addressing the vascular and immune limitations discussed above requires moving beyond avascular, immune‐free organoid design. Recent studies have responded by integrating vascular, genetic, biophysical, and microenvironmental components into PDGBOs to enhance physiological fidelity and predictive power for precision oncology — the specific strategies taken up in the sections below.

### 8.1 Vascularized Organoid Models for GBM Invasion

Incorporation of endothelial components into PDGBOs has emerged as a pivotal step toward clinically relevant invasion modelling. Glioblastoma‐cerebral organoid assembloids (GCOAs), pioneered by Wang et al. (2024) recapitulate patient‐specific invasive trajectories, tumor‐normal brain interactions, and transcriptionally distinct invasive subpopulations [67]. Complementary microfluidic vascular niche models developed by Truong et al. demonstrate that endothelial‐derived CXCL12‐CXCR4 signaling sustains GSC invasion, a process reversible with CXCR4 antagonists [229]. Similarly, collagen‐based vascular platforms show that endothelial cells selectively promote Nestin^+^ glioma‐initiating cell invasion via integrin‐dependent mechanisms [230, 231, 232]. Beyond invasion, co‐culture strategies integrating human endothelial cells with cerebral organoidsp or PDGBOs generate lumenized capillary‐like networks that improve oxygenation, nutrient diffusion, and long‐term tissue viability [233, 234]. Advanced BBB organoid systems incorporating endothelial cells, pericytes, astrocytes, and microglia reproduce tight junction integrity, selective permeability, and receptor‐mediated transcytosis, enabling realistic CNS drug delivery testing [65, 235]. VEGF‐driven vascular induction strategies further permit modelling of angiogenesis and resistance to anti‐angiogenic therapies. Vascularized PDGBOs enable realistic modelling of tumor invasion along vessels, hypoxia‐driven resistance, and heterogeneity in drug delivery‐factors that critically influence patient response but are invisible in avascular systems. Importantly, these models allow patient‐specific testing of anti‐angiogenic therapies, BBB‐penetrant drugs, and combination regimens under physiologically relevant perfusion conditions, improving prediction of treatment efficacy and failure [231].

### 8.2 Genetic Engineering of PDGBOs

Genetic engineering approaches further enhance organoid precision by embedding patient‐relevant oncogenic drivers within structured microenvironments. Studies by Shi et al. demonstrate that ETV2‐driven vasculogenic programming generates vascularized cortical organoids with BBB‐like features, enabling simultaneous interrogation of genotype‐vasculature interactions [236]. Engineered GBM organoids (eGBOs) developed by Ishahak et al. faithfully recapitulate early tumorigenesis, spatial hierarchy, and transcriptional states observed in patient tumors [237]. Notably, LEGO models introduced by Wang et al. integrate multi‐omics profiling to reveal mutation‐specific metabolic and phenotypic dependencies, such as NF1‐driven mesenchymal transitions and lipid rewiring. These platforms enable genotype‐informed drug screening that is not feasible in conventional organoids. eGBOs enable direct linkage between genotype and therapeutic vulnerability, allowing mutation‐informed drug screening and resistance modelling. Integration of multi‐omics readouts further supports identification of patient‐specific metabolic and signaling dependencies, advancing precision therapy beyond histology‐driven treatment paradigms [67, 238].

### 8.3 3D Bioprinting and Microfluidic Platforms

3D bioprinting and organ‐on‐chip technologies enable spatially controlled assembly of tumor, stromal, immune, and vascular compartments within biomimetic matrices. These systems reproduce perfusable vasculature, immune infiltration, oxygen gradients, and dynamic drug exposure, overcoming key limitations of static organoids. Bioprinted GBM models containing astrocytes, microglia, neural progenitors, and endothelial networks closely mirror patient‐derived transcriptional signatures and resistance phenotypes [239, 240]. Integration of computational tools such as GlioML further strengthens predictive capacity by linking organoid phenotypes to clinical outcomes [241]. These platforms allow realistic simulation of drug diffusion, immune infiltration, and adaptive resistance under treatment‐like conditions. When coupled with machine‐learning frameworks, they enable rapid, patient‐specific prediction of therapeutic response and optimization of combination strategies‐critical for clinical decision‐making in aggressive, fast‐progressing GBM.

### 8.4 Decellularized Brain ECM Platforms

To address biochemical and biomechanical limitations of Matrigel‐based systems, multiple groups have introduced decellularized brain ECM platforms that preserve native tissue architecture and signaling cues [242]. GBM cells cultured on dBECM scaffolds exhibit enhanced stemness, metabolic adaptation, ECM remodeling, and chemoresistance, more closely resembling in vivo tumors. ECM‐based hydrogels and slices developed by Wang et al. further support high‐resolution imaging and metabolic profiling. When combined with patient‐derived ECM, spatial mapping, and multi‐omics integration, these platforms provide a powerful route toward personalized modelling of invasion and therapy resistance. Embedding PDGBOs within native brain ECM better captures invasion, stemness, metabolic reprogramming, and chemoresistance observed in patients, enabling more faithful assessment of therapeutic efficacy. These platforms are particularly valuable for evaluating invasion‐targeting therapies and metabolism‐focused interventions that fail in conventional organoid systems [243].

Collectively, the studies summarized above demonstrate that incorporation of vascular, genetic, immune, and ECM components pioneered by multiple groups substantially improves the clinical relevance of PDGBOs. Patient‐derived GSC organoids are increasingly being applied to predict invasion, therapeutic resistance, and individualized drug response, with emerging clinical studies summarized in Table 4. Immune‐competent, vascularized, and genetically informed PDGBOs represent a necessary evolution toward functional precision oncology for GBM, rather than descriptive tumor modelling alone.

**TABLE 4:** Recent clinical studies integrating PDGBMOs for treatment response prediction.

Clinical Trial Title and ID	Study Location and Type	Patient Criteria	Objectives & Methods	Status	References
**Patient's Derived Organoids for Drug Screening in Glioblastoma (GlioPDO)** Official Title: Development and Characterization of Patient's Derived Organoids as a Platform for the Screening of Novel Therapeutic Treatments for Glioblastoma Multiforme ID: NCT06781372	Location: Fondazione Policlinico Universitario Agostino Gemelli IRCCS Type: Interventional	Adults (≥18) IDH‐wildtype GBM Undergoing resective neurosurgery Exclusions: needle biopsies, prior neoadjuvant chemo/RT, non‐GBM brain surgery	Generate PDOs from surgical tumor biopsies; establish neurosphere cultures. Apply epigenetic (DNMTi, HDACi) and splicing inhibitors (PRMT5i, SF3B1 targeting) to PDOs and GSCs. Investigate activation of transposable elements (LTR12C family) to produce immunogenic neoepitopes. Correlate in vitro drug responses with tumor molecular profiles and patient outcomes. Target: 100 patients.	Not Yet Recruiting	[244]
**Drug Response Testing and Repurposing Using Glioblastoma Organoid** Official Title: Patient‐derived Organoids as Predictive Models for Drug Response Testing and Repurposing in Glioblastoma Therapy ID: NCT06782984	Location: Chungnam National University Hospital, Daejeon, South Korea(multicenter: 5 tertiary hospitals) Type: Observational	Primary or recurrent GBM Treated with surgery + TMZ‐based chemoradiation Sufficient tumor sample for organoid culture Exclusions: no concurrent chemoradiation therapy (CCRT) post‐surgery, no post‐CCRT MRI, patient refusal	Evaluate whether patient‐derived GBO Drug Sensitivity Testing (GBO‐DST) recapitulates clinical response to TMZ. Establish 100 GBO lines from 150 patients using serum‐free protocol. Screen FDA‐approved drugs incl. Lazertinib (third‐gen EGFR inhibitor) and Regorafenib. Correlate GBO‐DST results with PFS and OS; assess multi‐kinase inhibitors and EGFR‐TKIs. Retrospective correlative design – no alteration of patient treatment regimens.	Active, Recruiting	[245]
**Patient‐Derived Glioma Stem Cell Organoids** Official Title: Glioma Stem Cell Organoids: Preclinical Model of Glioblastoma Heterogeneity to Explore Resistance Mechanisms to Conventional Treatment Schedules. ID: NCT04868396	Location: Maastricht Radiation Oncology, Netherlands Type: Observational	Adults (>18) MRI suggestive of GBM Exclusions: Karnofsky index < 70, clotting disorders, neurosurgical contraindications for gross total resection	Generate primary organoid cultures from GBM biopsy specimens. Investigate treatment resistance and aggressive tumor growth. Analyze MGMT heterogeneity and resistance to TMZ. Identify new therapies and biomarkers using exome sequencing, single‐cell proteomics, and IHC.	Active, Not Recruiting	[246]
**Glioblastoma Organoids (GBOs) as a Platform for Testing** Official Title: Feasibility of ORGAnoids in Routine Clinical Practice for Molecular Analysis of the GLIOvascular Niche in Patients with Primary Brain Tumors. ID: NCT07029100	Location: Centre Henri Becquerel, France (3 locations) Type: Interventional	Adults (≥18) with suspected high‐grade glioma (MRI‐based) undergoing tumor resection Eligible for standard adjuvant therapy (RT + TMZ ± TTFields) Exclusions: active/recent malignancy (<2 years), pregnancy	Establish feasibility of generating GBOs and blood vessel organoids (BVOs) from surgical samples Perform short‐term (≤7 days) molecular and phenotypic characterization Correlate organoid features with patient clinical outcomes and plasma biomarkers Evaluate organoid response alongside standard‐of‐care treatment.	Not Yet Recruiting	[247]
**Grafts of GSCs Into Brain Organoids for Testing Anti‐Invasion Drugs** Official Title: Grafts of Patient‐derived Glioblastoma Stem Cells on to Autologous Brain Organoids. A Precision Medicine Model for Testing Drugs Against Tumor Invasion ID: NCT05772741	Location: Fondazione Policlinico Universitario Agostino Gemelli IRCCS, Rome, Italy Type: Interventional	Adults (>18), Primary recurrence of GBM Exclusions: Insufficient tumor tissue for analysis	Grow GSC lines from patient tumor tissue. Form brain organoids from iPSCs to study brain invasion. Test gene and pharmacologic strategies to inhibit invasion and restore cilia. Use 3D co‐culture (GSC + brain organoid) to model and target invasive mechanisms.	Not Yet Recruiting	[248]

### 8.5 Preclinical Assessment of CAR‐T Cell Therapies in PDGBOs

A recurring problem in GBM immunotherapy trials is the time lag between treatment and measurable response. By the time MRI indicates whether a CAR‐T infusion has worked, the clinical window for adjustment has often closed. PDGBOs offer one way around this: ex vivo testing that runs alongside patient treatment rather than waiting for it to conclude. Recently, Logun et al. (2025) applied this approach directly within a phase 1 trial of intrathecal bivalent CAR‐T cells targeting EGFR and IL13Rα2 in recurrent GBM f(NCT05168423). GBOs were generated from resected tumor tissue within 2–3 weeks of surgery and co‐cultured with the same autologous CAR‐T products given to the corresponding patients, at a tumor cell‐to‐CAR‐T cell ratio of 10:1, timed to run concurrently with infusion. Tumor cytolysis was tracked continuously by impedance assay over 144 h and confirmed by cleaved caspase‐3 staining, with statistically significant killing in all six patient‐matched GBO sets. Early cytolysis at 24 h correlated with peak CAR‐T engraftment measured in patient CSF (r^2^ = 0.86, p = 0.0074), and the cytokine release profiles such as IFN‐γ, TNF‐α, and IL‐2 in co‐culture media were statistically indistinguishable from those in patient CSF through day 4. Post‐treatment flow cytometry of dissociated GBOs showed significant reductions in EGFR/IL13Rα2 double‐positive tumor cell populations, providing direct evidence of on‐target antigen depletion that could not be obtained clinically due to absence of post‐treatment tissue sampling. Importantly, GBO cytolysis data showed true anti‐tumor activity even when MRI results were unclear because of pseudoprogression [74].

However, a different but related challenge is that PDGBOs generated without immune components cannot model checkpoint biology or T cell exhaustion. More recently, Baisiwala et al. (2026) addressed this with the immune‐human organoid tumor transplantation (iHOTT) platform, which co‐transplants freshly dissociated patient GBM cells and matched peripheral blood mononuclear cells (PBMCs) into pre‐grown human cortical organoids at a 1:1 ratio, then maintains cultures for 7 days (Figure 3A). The system retained CD3+ T cells, B cells, and NK cells alongside tumor compartments. Tumor‐immune co‐culture induced IL‐6, IL‐8, IL‐10, and G‐CSF secretion not seen in tumor‐only or PBMC‐only controls (Figure 3B), and single‐cell analyses identified oligodendrocyte‐like tumor cells as dominant signaling senders with CD8+ T cells as primary receivers – patterns consistent with fresh patient tumor data. Pembrolizumab treatment in iHOTT reproduced the CD4+ T cell and B cell expansions, altered cell‐cell interaction networks, and TCR clonotype diversification observed in tumors from an independent 42‐patient pembrolizumab trial cohort (Figure 3C). Further, TCR clustering confirmed that clonal responses were predominantly patient‐specific, with minimal shared clonotypes across patients, which the authors interpret as consistent with the limited and variable clinical activity of PD‐1 blockade in GBM (Figure 3D) [249].

**FIGURE 3:**
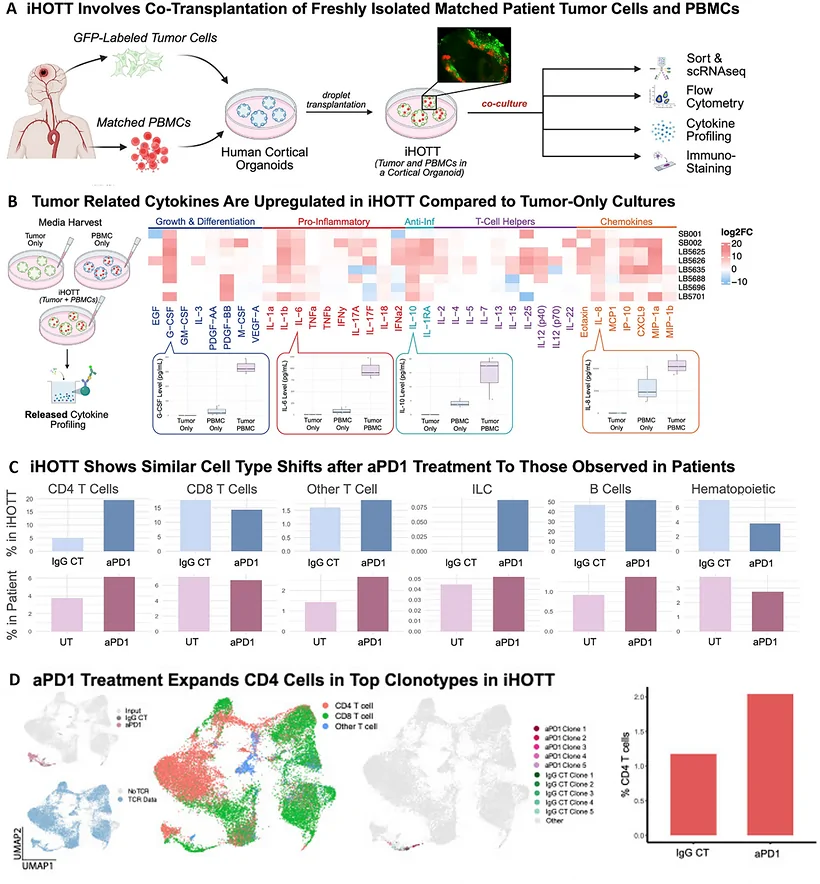
The iHOTT model for patient‐specific tumor‐immune co‐culture in GBM organoids. Panels reproduced from Baisiwala et al., Cell Reports 45, 116790 (2026) under Creative Commons Attribution‐NonCommercial 4.0 International License (CC BY‐NC 4.0; http://creativecommons.org/licenses/by‐nc/4.0/). (A) iHOTT model schematic: freshly dissociated GFP‐labelled patient GBM cells and matched PBMCs are co‐transplanted onto pre‐grown human cortical organoids by droplet transplantation and maintained for 7 days before multimodal analysis (scRNA‐seq, flow cytometry, cytokine profiling, immunostaining). (B) Cytokine profiling heatmap and representative boxplots showing upregulation of GBM‐associated cytokines G‐CSF, IL‐6, IL‐10, and IL‐8 in iHOTT (tumor + PBMC) co‐cultures relative to tumor‐only and PBMC‐only controls. Log2 fold change relative to tumor‐only controls shown. (C) Parallel bar plots comparing immune cell type composition (CD4+ T cells, CD8+ T cells, B cells, ILCs) between iHOTT treated with IgG control versus anti‐PD‐1 (aPD1/pembrolizumab), and between untreated and aPD1‐treated patient tumors, demonstrating concordant expansions. (D) UMAP and bar graph from TCR sequencing showing aPD1‐driven expansion of CD4+ T cell clonotypes in iHOTT, with top expanded clonotypes mapping predominantly to CD4+ T cell clusters (Reproduced from Logun et al. 2025 *Cell Stem Cell* 32, 181–190, and Baisiwala et al. 2026 *Cell Reports* 45, 116790 under the terms of the Creative Commons Attribution 4.0 International License (CC BY ‐ NC 4.0)).

What these two studies share is the conclusion that immune‐free organoid platforms are inadequate for evaluating T cell‐based therapies. Where they differ is in emphasis: Logun et al. showed that GBOs can function as real‐time avatars within an active trial, with cytolysis and cytokine readouts correlating directly with in vivo patient metrics; Baisiwala et al. showed that incorporating circulating immune cells enables mechanistic dissection of checkpoint resistance and TCR clonal dynamics at single‐cell resolution. Both capabilities are needed for GBM precision immunotherapy to move beyond empirical dosing toward patient‐stratified treatment selection.

## 9 Signaling Pathways in PDGBO Differentiation

For PDGBOs to achieve clinical relevance, signaling pathways must emerge endogenously within a 3D microenvironment rather than being inferred from 2D systems. The sections below focus on how signaling networks can be functionally reconstructed in PDGBOs to enhance precision modelling.

### 9.1 Importance of Signaling Pathways in PDGBOs

TCGA‐defined GBM subtypes – proneural, classical, and mesenchymal are driven by EGFR, PI3K/AKT/mTOR, NF‐κB, and STAT dependencies [250, 251], which PDGBOs retain, enabling functional stratification beyond genomics [252]. Co‐culture and vascularized PDGBO systems additionally allow paracrine/juxtacrine activation (Tie2, NF‐κB, GPCR) via endothelial and stromal interactions that tumor‐only cultures cannot recapitulate [253, 254], letting pathway activity arise dynamically from hypoxia, stiffness, and cell‐cell contact, and improving prediction of inhibitor and combination‐therapy response [108].

### 9.2 Key Signaling Pathways in PDGBOs

In vivo, this signaling depends on cross‐talk with vasculature, immune cells, and ECM largely absent from conventional organoids: PI3K–AKT–mTOR and RTK (EGFR, PDGFR) dysregulation governs TMZ/inhibitor response and adaptively rewires under hypoxic/endothelial influence to drive treatment failure [255, 256]. ‐ Notch and Wnt signaling are sustained within perivascular and matrix‐rich niches, preserving GSC plasticity and intratumoral heterogeneity that underlie recurrence. TGF‐β and N‐cadherin regulate invasion and radioresistance, whereas NF‐κB and HIF integrate immune suppression with metabolic adaptation [100, 257, 258]; lipid metabolism and HIF support survival under hostile conditions [259], while MYC drives tumorigenesis and NF‐κB drives immune evasion [260, 261]. Two studies show why this matters. Jacob et al. built PDGBOs from 53 patients and confirmed via multi‐omic profiling that organoids retained their source tumors' mutational makeup – with EGFR‐, NF1‐, and PI3K‐altered PDGBOs responding respectively to gefitinib, trametinib, and everolimus, mirroring patient‐specific genotypes that conventional cell lines lose during passaging. Bayin et al. showed Notch‐high GSCs localize to endothelium‐dependent perivascular zones while CD133‐high GSCs occupy hypoxic, glycolytic regions [262]. Preserving this therefore requires vascularized, immune‐competent, ECM‐rich PDGBOs [263, 264, 265].

### 9.3 Role of Growth Factors in PDGBOs

EGF/FGF2 sustain stem‐like, resistant states that can mask patient‐specific drug response unless carefully regulated [263, 266]; growth‐factor‐free systems instead reveal resistant SATB2^+^/HOPX^+^ progenitor populations [267]. Similarly, PDGF dependency stratifies responsiveness to PI3K–mTOR or PDGFR inhibition, while IDH1/2 and BRAF V600E mutations uncouple growth factor signaling from niche control, creating targetable vulnerabilities [74, 268, 269, 270]. Furthermore, hypoxia‐ and GDF15‐driven gradients integrate growth factor signaling with immune suppression, invasion, and radioresistance [263, 271, 272].

### 9.4 Techniques to Enhance Differentiation Efficiency in PDGBOs

Direct tumor‐derived PDGBOs retain native heterogeneity and resistance phenotypes, while iPSC‐engineered organoids enable controlled modelling of defined mutations [273, 274, 275]. Brain‐mimetic ECMs, physiological hypoxia, and co‐culture with endothelial, neural, and immune cells reproducibly induce invasion, pseudo‐palisading necrosis, and treatment‐relevant transcriptional states [276]; bioprinted GBM‐on‐chip, microfluidic perfusion, and decellularized ECM platforms extend this further by reconstructing nutrient gradients and chemoradiotherapy response [239, 277] – making differentiation strategy itself a variable that shapes predictive accuracy.

## 10 Technological Advancements and Future Prospects

As GBM organoid technologies mature, emerging analytical and computational tools are expanding their translational and clinical utility. Integrating biophysical methods, ML, and large‐scale organoid resources is increasingly critical for converting complex molecular and phenotypic data into clinically actionable insights, as illustrated in Figure 4. These advances position PDGBOs as functional platforms that bridge experimental modelling and patient‐specific therapeutic decision‐making.

**FIGURE 4:**
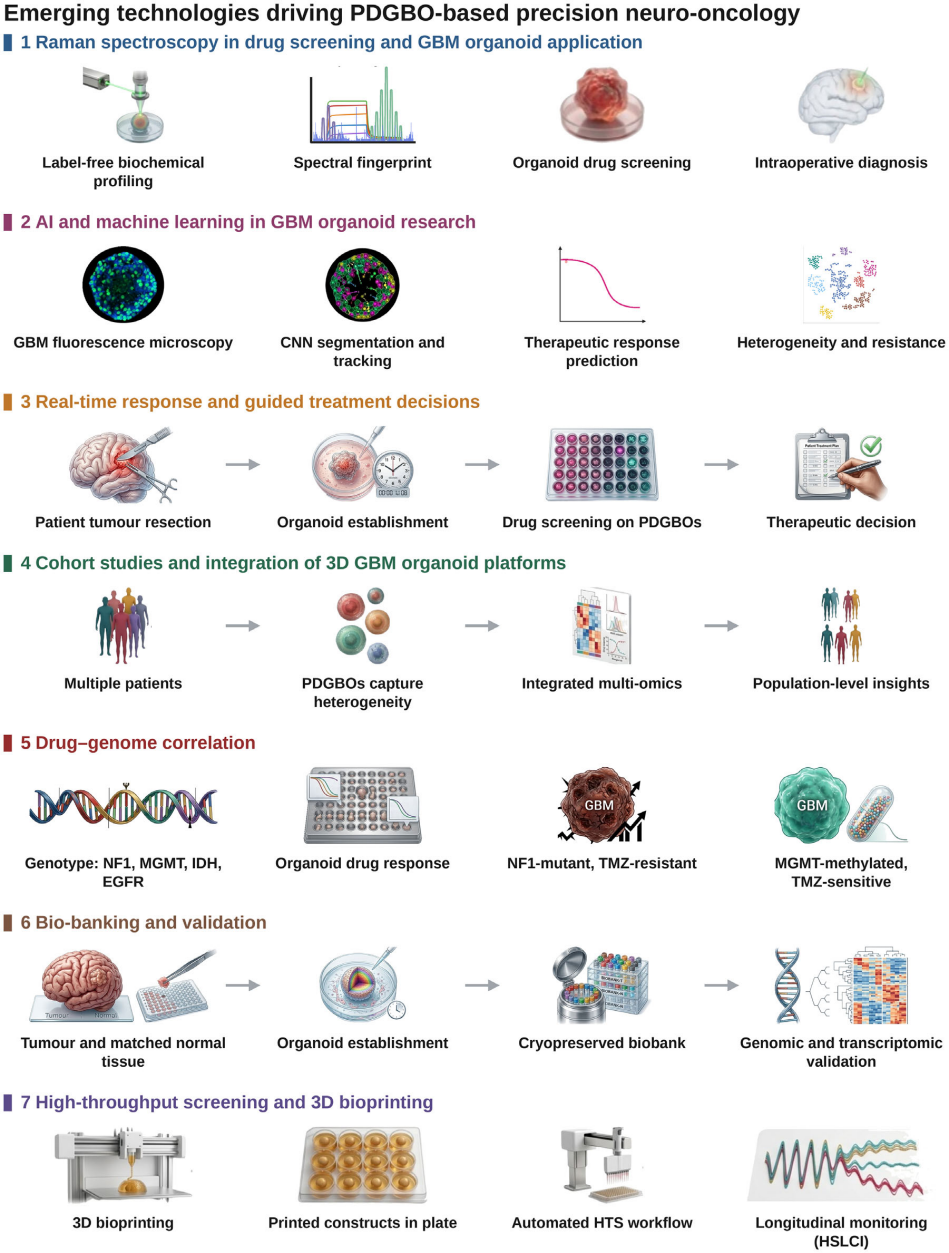
Integrated emerging technologies driving PDGBO‐based precision neuro‐oncology.

### 10.1 Raman Spectroscopy in Drug Screening and GBM Organoid Applications

Raman spectroscopy has emerged as a powerful, non‐invasive, label‐free modality for real‐time monitoring of biochemical states in PDGBOs [269]. By capturing vibrational signatures of proteins, lipids, nucleic acids, and ECM components, Raman profiling distinguishes differentiation states and drug‐treated versus untreated organoids, enabling high‐throughput screening in physiologically relevant 3D systems [277]. Importantly, Raman‐based approaches extend beyond in vitro modelling to clinical application. For instance, in fresh glioma biopsies, Raman spectroscopy combined with ML achieves high diagnostic accuracy in distinguishing tumor from normal tissue and identifying molecular biomarkers linked to malignancy [270]. A major advance is the Sentry System, which integrates Raman spectroscopy with support vector machine algorithms. In 67 patients with GBM, metastases, or meningiomas, ∼976 spectra were compared to histopathology. Diagnostic performance was strong: 91% sensitivity/specificity for GBM, 98%/96% for metastases, and 96%/96% for meningiomas. Protein‐ and lipid‐associated peaks enabled discrimination. Real‐time feedback enhances surgical precision and minimizes neurological damage [278]. Together, Raman spectroscopy with organoid platforms supports drug discovery and intraoperative decision‐making, advancing precision neuro‐oncology. Raman spectroscopy offers a non‐invasive, label‐free method to monitor biochemical changes in PDGBOs and distinguish drug responses in real time, making it ideal for high‐throughput drug screening. Combined with ML, it enables accurate intraoperative glioma detection and tumor margin delineation, improving surgical precision and patient outcomes. Integrating Raman spectroscopy with organoid platforms and AI‐driven data analysis advances personalized neuro‐oncology by enabling rapid, data‐rich functional assessment of treatment efficacy [279]. As biophysical imaging tools enhance our understanding to characterize organoids in real time, parallel advances in computational technologies are transforming how these complex datasets are interpreted and translated into clinical predictions. This shift toward automated, data‐driven analysis has positioned AI and ML as essential complements to organoid‐based platforms.

### 10.2 AI and Machine Learning in GBM Organoid Research

AI and ML are transforming GBM organoid research, automating morphology assessment and enabling high‐throughput drug screening [271, 280]. Image‐based phenotypic profiling is a key application: convolutional neural networks (CNNs) now automate segmentation, classification, and temporal tracking of 3D organoids, reducing bias and boosting throughput. Bian et al. (2021) demonstrated this directly, developing a deep learning framework for real‐time monitoring of organoid growth and drug responses, eliminating the need for labor‐intensive manual annotation [281]. AI also integrates multi‐omics datasets like genomic, transcriptomic, and proteomic, providing insight into heterogeneity, drug resistance, and tumor progression [272]. ML‐based studies have identified PKCδ and DNA‐PK as master kinases in mesenchymal‐like GBM, suggesting new therapeutic targets [282]. Predictive applications that link organoid AI readouts directly to individualized therapy remain at an earlier‐stage. Mestrallet et al. (2024) reported 82% accuracy in forecasting PD‐1 inhibitor responses using immune profiles from PDGBOs [283] – a proof‐of‐concept result rather than a clinically validated tool, and one that will need replication across larger, independent cohorts before individualized treatment decisions can be based on it. Realizing this translational potential also faces concrete barriers: biological variability between organoid batches, limited dataset sizes, the “black box” nature of deep learning models, and ethical concerns over patient‐derived data. With these caveats in mind, integrating AI with PDGBOs represents a promising future path toward real‐time, patient‐specific therapeutic decision‐making that could bridge experimental models and clinical neuro‐oncology [284, 285].

Alongside AI‐driven phenotypic analysis, understanding how genomic alterations influence drug sensitivity remains equally important, motivating the surge in drug‐genome interaction studies using organoid platforms discussed next.

### 10.3 Drug‐Genome Correlation Studies

GBM organoid technology clinically transforms precision oncology by directly linking tumor genetics to personalized treatment responses. For example, NF1‐mutant organoids, which display mesenchymal transcriptional signatures and TMZ resistance, highlight why patients with these mutations may respond poorly to standard chemotherapy, guiding clinicians to consider alternative or combination therapies targeting lipid metabolism or JNK signaling pathways. Conversely, MGMT‐methylated organoids' sensitivity to TMZ supports continued use of this frontline drug in these patients. By incorporating comprehensive multi‐omics data into genotype‐specific drug atlases, clinicians can stratify patients more accurately, select effective drug combinations, and predict treatment failures early. PDGBOs thus serve as functional avatars for real‐time therapeutic testing, enabling adaptive, individualized treatments and improving clinical outcomes in GBM management [67, 261]. As these genomic and pharmacological insights continue to accumulate, the need for standardized and renewable patient‐derived resources has driven the development of large‐scale organoid biobanks, which serve as the foundation for cross‐cohort translational research.

### 10.4 Bio‐Banking and Validation

GBM organoid biobanks are essential for translational research and precision oncology, preserving PDGBOs alongside matched normal tissue to study tumor heterogeneity, treatment resistance, and therapy response [275]. A biobank of 53+ patient samples showed organoids retain invasive phenotypes and genetic landscapes, enabling personalized therapy screening, including CAR‐T and immune checkpoint responses correlating with mutational and transcriptional profiles [48]. PDGBOs maintain key histopathological and genomic features, such as Ki67, CDX2, and β‐catenin, across passages, supporting reliable modelling of disease progression and targeted interventions [276]. High‐throughput drug screens revealed potent compounds, like tubulin inhibitors, alongside inter‐patient variability, highlighting the need for individualized approaches [113].

Large‐scale initiatives, including the Glioma Longitudinal Atlas Project and GLASS consortium, combine organoid biobanking with multi‐region profiling, enabling high‐resolution studies of evolution, heterogeneity, and resistance [286, 287]. These platforms provide standardized, renewable resources for integrated drug testing, molecular profiling, and therapy validation, advancing patient‐specific precision medicine. With validated biobanks supporting rapid organoid generation and testing, the next logical step is to translate these platforms into real‐time treatment decision‐guiding for individual patients [275].

### 10.5 Real‐Time Response and Guided Treatment Decisions

PDGBOs retain histopathological, molecular, and functional heterogeneity, enabling real‐time, patient‐specific therapy guidance. For instance, Loong et al. (2020) generated organoids from a 58‐year‐old patient, revealing shared alterations in primary and recurrent tumors, including PI3K/AKT/mTOR hyperactivation. Organoids confirmed TMZ resistance but sensitivity to cobimetinib and everolimus; everolimus treatment reduced tumor and edema, with disease worsening during treatment breaks and regression upon re‐initiation, illustrating PTEN‐pathway dependency and dynamic dosing guidance [288]. Across subtypes, organoid drug screening guided therapy: BRAF V600E PDGBOs identified vemurafenib plus trametinib as highly effective, achieving complete regression sustained over 13 months, while mutant IDH low‐grade glioma organoids confirmed TMZ responsiveness [289]. Rapid post‐surgery organoid establishment (≤15 days) enables timely drug selection, identifying agents like crizotinib, foretinib, and afatinib. Limitations such as BBB penetration and bypass pathway activation emphasize the need for combinatorial strategies to improve therapeutic durability [60]. To meet the demands of rapid clinical transition, and large‐scale drug screening, technological innovations such as 3D bioprinting now provide scalable platforms for high‐throughput assays.

### 10.6 High‐Throughput Screening With 3D Bioprinting

High‐throughput screening (HTS) approaches adapted to 3D bioprinted PDGBOs enable scalable and standardized assessment of therapeutic response. Drop‐on‐demand printing with alginate or Matrigel‐based bioinks supports high cell viability and reproducible organoid formation, allowing controlled evaluation of drug penetration and dose‐response relationships [290]. Notably, larger organoids exhibit increased IC_50_ values, reflecting clinically relevant diffusion‐limited resistance. Automated workflows, including robotic handling and multi‐well formats, facilitate parallel generation of PDGBOs for drug screening and molecular profiling while minimizing operator variability [291]. Integration of label‐free readouts, such as high‐speed live‐cell interferometry (HSLCI), enables longitudinal monitoring of individual organoids and identification of resistant subpopulations not captured by bulk viability assays. Together, HTS‐compatible bioprinted organoid platforms support population‐scale functional testing and strengthen the translational pipeline toward patient‐stratified therapy selection [292].

### 10.7 Cohort Studies and Integration of 3D GBM Organoid Platforms

Integrating multi‐patient cohort studies with PDGBOs is transforming translational neuro‐oncology by providing robust platforms for patient‐specific therapy interrogation and prospective drug stratification. PDGBOs reliably preserve the histopathological, genetic, and transcriptional heterogeneity of their donor tumors, including complex pharmacogenomic profiles and intratumoral diversity. This high‐fidelity retention enables individualized prediction of treatment responses and simulates real‐time tumor‐drug interactions beyond conventional cell line models [293]. Recent cohort studies have demonstrated genetic stability across multiple passages and confirmed individualized TMZ responses in organoids derived from distinct patients. Adaptive resistance mechanisms, such as JNK pathway upregulation, were elucidated through transcriptomic analyses, and targeted JNK inhibition was shown to synergistically enhance TMZ‐induced cytotoxicity. These findings directly position PDGBOs as both functional avatars and mechanistic discovery platforms, leveraging their dynamic response signatures to guide combination therapy design [294]. Parallel integration with HTS pipelines and biobanked organoid libraries enables simultaneous evaluation of hundreds of therapeutic agents across diverse patient ‘avatars’, opening avenues for real‐time therapy guidance and population‐level response simulation. Stratification of responders versus non‐responders is now feasible via genomic and transcriptomic biomarker signatures, facilitating the design of prospective drug stratification trials and informing cohort‐based precision treatment paradigms [241, 295].

Innovations in organoid engineering including microfluidic vascularization and immune cell incorporation have further improved model microenvironment fidelity, thus more accurately recreating human GBM angiogenesis and immune architecture. These advances allow for interrogation of drug penetration, immune evasion, and the impact of combinatorial immunotherapies, further bridging the translational gap [296]. The convergence of cohort‐scale organoid data, multidimensional ‐omics, and advanced functional platforms enables not only real‐time individualized therapy guidance but also discovery of new therapeutic biomarkers and repurposing strategies, moving GBM research decisively from descriptive to functional precision oncology [297].

## 11 Conclusion

PDGBOs have matured from descriptive experimental constructs into biologically faithful platforms for precision oncology. This review has traced that progression across two dimensions that are typically treated in isolation: the engineering of physiological fidelity – vasculature, immune compartments, and brain‐mimetic architecture, and the computational layer of AI/ML that converts organoid data into predictive, patient‐specific insight. Neither dimension alone is sufficient; their convergence is what will determine whether organoid platforms remain a research tool or become a clinical instrument.

### 11.1 Addressing Research Gaps and Emerging Trends: Paving the Way Forward

The biology is understood. The models exist. What the field now lacks are the infrastructure, integration, and analytical rigor to make organoid‐based research clinically actionable. With that in mind, three priorities stand above the rest. The first is vascular and immune integration. Avascular, immune‐free organoids cannot model the perivascular GSC niche, TAM‐driven resistance, or checkpoint biology – the very mechanisms that explain why GBM therapies fail. Next‐generation platforms must incorporate perfusable endothelial networks, patient‐matched myeloid and lymphoid components, and the BCSFB interface to extend organoid utility to CSF‐pathway drug delivery and immune trafficking. The second priority is standardization. High organoid variability, inconsistent establishment rates, and absence of quality control metrics remain barriers to reproducibility and clinical adoption. Automated bioprinting, defined ECM formulations, and population‐scale functional screening pipelines are needed to generate data with the consistency clinical translation demands. The third priority, and the most novel, is AI and ML integration. Cohort‐scale organoid biobanks combined with multi‐omics profiling and ML can enable real‐time patient stratification, resistance trajectory prediction, and adaptive trial design. This is not incremental progress; it represents a fundamental shift in how organoid data is used. When a patient's tumor drives an organoid platform whose drug response profile is interpreted by a validated AI model and fed directly into a treatment decision, GBM research will have truly bridged the gap between the laboratory and the clinic.

## Author Contributions

**Sandbhor Puja**: conceptualization, investigation, funding acquisition, writing – original draft, methodology, validation, visualization, writing – review and editing, formal analysis, project administration, data curation, supervision, resources. **Arun Bhavyadharshini**: investigation, writing – original draft, methodology, validation, visualization, writing – review and editing, formal analysis, data curation. **John Geofrey**: writing – original draft, methodology, validation, visualization, writing – review and editing, formal analysis, data curation. **Mudliyar Siddharth**: writing – original draft, methodology, validation, visualization, writing – review and editing, formal analysis, data curation. **Ghanekar Shubham**: writing – original draft, methodology, validation, visualization, writing – review and editing, formal analysis, data curation. **Moyiadi Aliasgar**: project administration, resources, supervision, writing – review and editing. **Shetty Prakash**: project administration, resources, formal analysis, writing – review and editing. **Singh Vikas**: project administration, resources, formal analysis, writing – review and editing. **Sadwani Nidhisha**: project administration, resources, writing – review and editing. **Garg Akshohini**: project administration, resources, writing – review and editing. **Sahay Ayushi**: writing – review and editing, project administration, resources. **Chatterjee Abhishek**: writing – review and editing, formal analysis, project administration, supervision, resources. **Epari Sridhar**: project administration, resources, writing – review and editing. **Gupta Tejpal**: writing – review and editing, project administration, resources. **Dasgupta Archya**: writing – review and editing, project administration, resources. **Goda Jayant**: writing – review and editing, project administration, formal analysis, resources, funding acquisition.

## Funding

Although no specific funding was received for the preparation of this review, the scientific viewpoints and experience synthesized in this article have been informed by research supported through the Inspire Faculty Fellowship, Department of Science and Technology (DST), Ministry of Science and Technology, New Delhi, India (IFA 23‐ENG 359), and the Tata Memorial Hospital Intramural Research Grant (IEC project No. 901082) and the Prime Minister Early Career Research Fellowship supported by Anusandhan National Research Foundation (ANRF) program (Grant No. ANRF/ECRG/2024/001010/ENS) by the Dept of Science and Technology, New Delhi, India. The PS sincerely acknowledges this support.

## Conflicts of Interest

The authors declare no conflicts of interest

## Data Availability

The data that support the findings of this study are available from the corresponding author upon reasonable request.

## References

[1] T. J. Abou‐Antoun , J. S. Hale , J. D. Lathia , and S. M. Dombrowski , “Brain Cancer Stem Cells in Adults and Children: Cell Biology and Therapeutic Implications,” Neurotherapeutics 14, no. 2 (2017): 372–384, 10.1007/s13311-017-0524-0. PMC5398995 28374184

[2] X. Li , C. Li , W. Zhang , Y. Wang , P. Qian , and H. Huang , “Inflammation and aging: Signaling pathways and intervention therapies,” Signal Transduction and Targeted Therapy 8, no. 1 (2023): 239, 10.1038/s41392-022-01260-z. PMC10248351 37291105

[3] R. Azzarelli , B. D. Simons , and A. Philpott , “The Developmental Origin of Brain Tumours: A Cellular and Molecular Framework Development,” The Company of Biologists 145 (2018): dev162693, https://journals.biologists.com/dev/article/145/10/dev162693/48541/The‐developmental‐origin‐of‐brain‐tumours‐a. 10.1242/dev.162693 PMC6001369 29759978

[4] “Brain Metastases | Radiology Reference Article | Radiopaedia.Org.” Accessed: May 04, 2026. [Online]. Available: https://radiopaedia.org/articles/brain‐metastases?lang=us.

[5] C. Xu , P. Hou , X. Li , et al., “Comprehensive Understanding of Glioblastoma Molecular Phenotypes: Classification, Characteristics, and Transition,” Cancer Biology & Medicine (2024): 20230510, https://www.cancerbiomed.org/content/early/2024/05/06/j.issn.2095‐3941.2023.0510. 10.20892/j.issn.2095-3941.2023.0510 PMC11131044 38712813

[6] Y. W. Park , P. Vollmuth , M. Foltyn‐Dumitru , et al., “The 2021 WHO Classification for Gliomas and Implications on Imaging Diagnosis: Part 1—Key Points of the Fifth Edition and Summary of Imaging Findings on Adult‐Type Diffuse Gliomas,” Journal of Magnetic Resonance Imaging 58, no. 3 (2023): 677–689, 10.1002/jmri.28743. 37069792

[7] M. Price , C. Ballard , J. Benedetti , et al., “CBTRUS Statistical Report: Primary Brain and Other Central Nervous System Tumors Diagnosed in the United States in 2017–2021,” Neuro‐Oncology Oxford Academic 26 (2024): vi1–vi85, https://academic.oup.com/neuro‐oncology/article/26/Supplement_6/vi1/7797290. 10.1093/neuonc/noae145 PMC11456825 39371035

[8] C. Hertler , J. Felsberg , D. Gramatzki , et al., “Long‐term Survival With IDH Wildtype Glioblastoma: First Results From the ETERNITY Brain Tumor Funders′ Collaborative Consortium (EORTC 1419),” European Journal of Cancer 189 (2023): 112913, 10.1016/j.ejca.2023.05.002. 37277265

[9] “Glioblastoma Prognosis ,” The Brain Tumour Charity. Accessed: May 04, 2026. [Online]. Available: https://www.thebraintumourcharity.org/brain‐tumour‐diagnosis‐treatment/types‐of‐brain‐tumour‐adult/glioblastoma/glioblastoma‐prognosis/.

[10] B. K. Ahir , H. H. Engelhard , and S. S. Lakka , “Tumor Development and Angiogenesis in Adult Brain Tumor: Glioblastoma,” Molecular Neurobiology 57 (2020): 2461–2478, https://link.springer.com/article/10.1007/s12035‐020‐01892‐8. 10.1007/s12035-020-01892-8 PMC7170819 32152825

[11] K. Gollapalli , S. Ghantasala , S. Kumar , et al., “Subventricular Zone Involvement in Glioblastoma—A Proteomic Evaluation and Clinicoradiological Correlation,” Scientific Reports 7 (2017): 1449, https://www.nature.com/articles/s41598‐017‐01202‐8. 10.1038/s41598-017-01202-8 PMC5431125 28469129

[12] J. Beiriger , A. Habib , N. Jovanovich , et al., “The Subventricular Zone in Glioblastoma: Genesis, Maintenance, and Modeling,” Frontiers in Oncology 12 (2022): 790976, 10.3389/fonc.2022.790976. PMC8960165 35359410

[13] T. Oishi , S. Koizumi , and K. Kurozumi , “Molecular Mechanisms and Clinical Challenges of Glioma Invasion,” Brain Sciences 12, no. 2 (2022): 291, 10.3390/brainsci12020291. PMC8870089 35204054

[14] E. De Fazio , M. Pittarello , A. Gans , et al., “Intrinsic and Microenvironmental Drivers of Glioblastoma Invasion,” International Journal of Molecular Sciences 25, no. 5 (2024): 2563, 10.3390/ijms25052563. PMC10932253 38473812

[15] J. Khalifa , F. Tensaouti , A. Lusque , et al., “Subventricular Zones: New Key Targets for Glioblastoma Treatment,” Radiation Oncology 12 (2017): 67, https://link.springer.com/article/10.1186/s13014‐017‐0791‐2. 10.1186/s13014-017-0791-2 PMC5397708 28424082

[16] Y.‐F. Huang , M.‐T. Chiao , T.‐H. Hsiao , et al., “Genetic Mutation Patterns Among Glioblastoma Patients in the Taiwanese Population—Insights From a Single Institution Retrospective Study,” Cancer Gene Therapy 31, no. 6 (2024): 894–903, 10.1038/s41417-024-00746-y. PMC11192627 38418842

[17] S. Han , Y. Liu , S. J. Cai , et al., “IDH Mutation in Glioma: Molecular Mechanisms and Potential Therapeutic Targets,” British Journal of Cancer 122, no. 11 (2020): 1580–1589, 10.1038/s41416-020-0814-x. PMC7250901 32291392

[18] S. Grochans , A. M. Cybulska , D. Siminska , et al., “Epidemiology of Glioblastoma Multiforme–Literature Review,” Cancers 14, no. 10 (2022): 2412, 10.3390/cancers14102412. PMC9139611 35626018

[19] F. Hanif , K. Muzaffar , K. Perveen , S. M. Malhi , and S. U. Simjee , “Glioblastoma Multiforme: A Review of Its Epidemiology and Pathogenesis Through Clinical Presentation and Treatment,” Asian Pacific Journal of Cancer Prevention 18, no. 1 (2017): 3–9, 10.22034/APJCP.2017.18.1.3. PMC5563115 28239999

[20] A. Shergalis , A. Bankhead , U. Luesakul , N. Muangsin , and N. Neamati , “Current Challenges and Opportunities in Treating Glioblastoma,” Pharmacological Reviews 70, no. 3 (2018): 412–445, 10.1124/pr.117.014944. PMC5907910 29669750

[21] D. Saha , S. D. Rabkin , and R. L. Martuza , “Temozolomide Antagonizes Oncolytic Immunovirotherapy in Glioblastoma,” Journal for ImmunoTherapy of Cancer 8, no. 1 (2020): 000345, 10.1136/jitc-2019-000345. PMC7252967 32457126

[22] M. Fu , Z. Zhou , X. Huang , et al., “Use of Bevacizumab in Recurrent Glioblastoma: A Scoping Review and Evidence Map,” BMC cancer 23, no. 1 (2023): 544, 10.1186/s12885-023-11043-6. PMC10265794 37316802

[23] R. S. Angom , N. M. R. Nakka , and S. Bhattacharya , “Advances in Glioblastoma Therapy: An Update on Current Approaches,” Brain Sciences 13, no. 11 (2023): 1536, 10.3390/brainsci13111536. PMC10669378 38002496

[24] I. Ntafoulis , S. L. W. Koolen , S. Leenstra , and M. L. M. Lamfers , “Drug Repurposing, a Fast‐Track Approach to Develop Effective Treatments for Glioblastoma,” Cancers 14, no. 15 (2022): 3705, 10.3390/cancers14153705. PMC9367381 35954371

[25] B. J. Neves , J. P. Agnes , M. D. N. Gomes , et al., “Efficient Identification of Novel Anti‐Glioma Lead Compounds by Machine Learning Models,” European Journal of Medicinal Chemistry 189 (2020): 111981, 10.1016/j.ejmech.2019.111981. 31978780

[26] A. Majchrzak‐Celińska and E. Studzińska‐Sroka , “New Avenues and Major Achievements in Phytocompounds Research for Glioblastoma Therapy,” Molecules 29, no. 7 (2024): 1682, 10.3390/molecules29071682. PMC11013944 38611962

[27] K. Zhai , M. Siddiqui , B. Abdellatif , A. Liskova , P. Kubatka , and D. Büsselberg , “Natural Compounds in Glioblastoma Therapy: Preclinical Insights, Mechanistic Pathways, and Outlook,” Cancers 13, no. 10 (2021): 2317, 10.3390/cancers13102317. PMC8150927 34065960

[28] B. C. Das , J. J. Lepe , M. Adil Shareef , N. Lomeli , S. Das , and D. A. Bota , “Identification of New Hit to Lead magmas Inhibitors as Potential Therapeutics for Glioblastoma,” Bioorganic & Medicinal Chemistry Letters 91 (2023): 129330, 10.1016/j.bmcl.2023.129330. PMC10506439 37201660

[29] K. Duval , H. Grover , L.‐H. Han , et al., “Modeling Physiological Events in 2D vs. 3D Cell Culture,” Physiology 32 (2016): 2016, https://journals.physiology.org/doi/10.1152/physiol.00036.2016. 10.1152/physiol.00036.2016 PMC5545611 28615311

[30] M. Paolillo , S. Comincini , and S. Schinelli , “In Vitro Glioblastoma Models: A Journey Into the Third Dimension,” Cancers 13, no. 10 (2021): 2449, 10.3390/cancers13102449. PMC8157833 34070023

[31] J. Park , I. Koh , J. Cha , et al., “Comparison of Glioblastoma Cell Culture Platforms Based on Transcriptional Similarity With Paired Tissue,” Pharmaceuticals 17, no. 4 (2024): 529, 10.3390/ph17040529. PMC11054899 38675489

[32] C. Meulenbroeks , P. Stathi , M. van de Wetering , et al., “Mdb‐68. DISCOVERING Drug Sensitivities in Medulloblastoma Using Patient‐ And Pdx‐Derived Tumoroids,” Neuro‐Oncology 26, no. 4 (2024): 0, 10.1093/neuonc/noae064.517.

[33] C. Xie , G. Jobin , H. Mendez‐Gomez , et al., “1416 A Patients‐Derived Glioblastoma Tumoroids Model to Study Individual Response to Chemo‐Radiation,” Journal for ImmunoTherapy of Cancer (2024): A1583, https://jitc.bmj.com/content/12/Suppl_2/A1583.

[34] J. Alcaniz , M. Becker , S. Krassnig , et al., “Clinically Relevant Glioblastoma Patient‐derived Xenograft Models to Guide Drug Development and Identify Molecular Signatures,” Frontiers (2023): 1129627, https://www.frontiersin.org/journals/oncology/articles/10.3389/fonc.2023.1129627/full. 10.3389/fonc.2023.1129627 PMC10126369 37114125

[35] A. Pliakopanou , I. Antonopoulos , N. Darzenta , et al., “Glioblastoma Research on Zebrafish Xenograft Models: A Systematic Review,” Clinical and Translational Oncology 26 (2024): 311–325, https://link.springer.com/article/10.1007/s12094‐023‐03258‐7. 10.1007/s12094-023-03258-7 PMC10810942 37400666

[36] R. Mair , F. Mouliere , C. G. Smith , et al., “Measurement of Plasma Cell‐Free Mitochondrial Tumor DNA Improves Detection of Glioblastoma in Patient‐Derived Orthotopic Xenograft Models,” Cancer Research 79 (2019): 220–230, https://aacrjournals.org/cancerres/article/79/1/220/633152/Measurement‐of‐Plasma‐Cell‐Free‐Mitochondrial. 10.1158/0008-5472.CAN-18-0074 PMC6753020 30389699

[37] W. Liu , Y. Cui , X. Zheng , K. Yu , and G. Sun , “Application Status and Future Prospects of the PDX Model in Lung Cancer,” Frontiers In Oncology 13 (2023): 1098581, 10.3389/fonc.2023.1098581. PMC10080030 37035154

[38] B. Pan , X. Wei , and X. Xu , “Patient‐derived Xenograft Models in Hepatopancreatobiliary Cancer,” Cancer Cell International 22, no. 1 (2022): 41, 10.1186/s12935-022-02454-9. PMC8796540 35090441

[39] S. Murugappan , A. Mali , and N. Thorat , “Glioblastoma‐Neuroblastoma co‐cultured Multicellular Spheroid Model for Anti‐cancer Drug Screening,” bioRxiv (2025): 622957, 10.1101/2024.11.11.622957.

[40] J. Alcaniz , L. Winkler , M. Dahlmann , et al., “Clinically Relevant Glioblastoma Patient‐derived Xenograft Models to Guide Drug Development and Identify Molecular Signatures,” Frontiers in Oncology 13 (2023): 1129627, 10.3389/fonc.2023.1129627. PMC10126369 37114125

[41] N. Yadav and B. W. Purow , “Understanding Current Experimental Models of Glioblastoma‐Brain Microenvironment Interactions,” Journal of Neuro‐Oncology 166, no. 2 (2024): 213–229, 10.1007/s11060-023-04536-8. PMC11056965 38180686

[42] M. Logun , W. Zhao , L. Mao , and L. Karumbaiah , “Microfluidics in Malignant Glioma Research and Precision Medicine,” Advanced Biosystems 2, no. 5 (2018): 1700221, 10.1002/adbi.201700221. PMC5959050 29780878

[43] C. Bayona , C. Olaizola‐Rodrigo , V. Sharko , et al., “A Novel Multicompartment Barrier‐Free Microfluidic Device Reveals the Impact of Extracellular Matrix Stiffening and Temozolomide on Immune‐Tumor Interactions in Glioblastoma,” Small 21, no. 9 (2025): 2409229, 10.1002/smll.202409229. 39901479

[44] J. M. Ayuso , R. Monge , A. Martínez‐González , et al., “Glioblastoma on a Microfluidic Chip: Generating Pseudopalisades and Enhancing Aggressiveness Through Blood Vessel Obstruction Events,” Neuro‐Oncology 19, no. 4 (2017): 503–513, 10.1093/neuonc/now230. PMC5464359 28062831

[45] L. Neufeld , E. Yeini , N. Reisman , et al., “Microengineered Perfusable 3D‐bioprinted Glioblastoma Model for in Vivo Mimicry of Tumor Microenvironment,” Science Advances 7, no. 34 (2021): abi9119, 10.1126/sciadv.abi9119. PMC8373143 34407932

[46] M. A. Marques‐Torrejon , E. Gangoso , and S. M. Pollard , “Modelling Glioblastoma Tumour‐host Cell Interactions Using Adult Brain Organotypic Slice co‐culture,” Disease Models & Mechanisms 11, no. 2 (2018): dmm031435, 10.1242/dmm.031435. PMC5894940 29196443

[47] I. Zhang , P. Lépine , C. Han , et al., “Nanotherapeutic Modulation of Human Neural Cells and Glioblastoma in Organoids and Monocultures,” Cells 9, no. 11 (2020): 2434, 10.3390/cells9112434. PMC7695149 33171886

[48] F. Jacob , R. D. Salinas , D. Y. Zhang , et al., “A Patient‐Derived Glioblastoma Organoid Model and Biobank Recapitulates Inter‐ and Intra‐tumoral Heterogeneity,” Cell 180, no. 1 (2020): 188–204.e22, 10.1016/j.cell.2019.11.036. PMC7556703 31883794

[49] L. T. Rodgers , J. L. Villano , A. M. S. Hartz , and B. Bauer , “Glioblastoma Standard of Care: Effects on Tumor Evolution and Reverse Translation in Preclinical Models,” Cancers 16, no. 15 (2024): 2638, https://www.mdpi.com/2072‐6694/16/15/2638. 10.3390/cancers16152638 PMC11311277 39123366

[50] C.‐H. Chen , R. L. Chin , G. P. Hartley , et al., “Novel Murine Glioblastoma Models That Reflect the Immunotherapy Resistance Profile of a human Disease,” Neuro‐Oncology 25, no. 8 (2023): 1415–1427, 10.1093/neuonc/noad025. PMC10398813 36705543

[51] B. J. Umlauf and E. V. Shusta , “Exploiting BBB Disruption for the Delivery of Nanocarriers to the Diseased CNS,” Current Opinion in Biotechnology 60 (2019): 146–152, 10.1016/j.copbio.2019.01.013. PMC6728237 30849699

[52] K. E. Blethen , T. A. Arsiwala , R. A. Fladeland , et al., “Modulation of the Blood‐Tumor Barrier to Enhance Drug Delivery and Efficacy for Brain Metastases,” Neuro‐Oncology Advances Oxford Academic 3 (2021): v133–v143, https://academic.oup.com/noa/article/3/Supplement_5/v133/6444211. 10.1093/noajnl/vdab123 PMC8633736 34859240

[53] C. He , Z. Wu , M. Zhuang , et al., “Focused Ultrasound‐Mediated Blood‐Brain Barrier Opening Combined With Magnetic Targeting Cytomembrane Based Biomimetic Microbubbles for Glioblastoma Therapy,” Journal of Nanobiotechnology 21 (2023): 297, https://link.springer.com/article/10.1186/s12951‐023‐02074‐z. 10.1186/s12951-023-02074-z PMC10463668 37626360

[54] X. Chen , Y. Chen , X. Chen , et al., “Single‐cell RNA Sequencing Reveals Intra‐tumoral Heterogeneity of Glioblastoma and a Pro‐tumor Subset of Tumor‐associated Macrophages Characterized by *EZH2* Overexpression,” Biochimica et Biophysica Acta (BBA)—Molecular Basis of Disease 1868, no. 12 (2022): 166534, 10.1016/j.bbadis.2022.166534. 36057370

[55] B. J. Erickson and E. Galanis , “Where Size Matters: Imaging‐based Biomarkers for Patient Stratification,” Neuro‐Oncology 19, no. 1 (2017): 7–8, 10.1093/neuonc/now248. PMC5193032 28031381

[56] A. Yekula , R. R. Kitchen , S. K. Chakrabortty , et al., “OMRT‐2. Liquid Biopsy for Patient Stratification and Monitoring of dacomitinib Clinical Trial in Patients With EGFR Amplified Recurrent Glioblastoma,” Neurooncology Advances 3, no. 2 (2021): ii7, 10.1093/noajnl/vdab070.028.

[57] J. M. Bark , A. Kulasinghe , B. Chua , B. W. Day , and C. Punyadeera , “Circulating Biomarkers in Patients With Glioblastoma,” British Journal of Cancer 122 (2020): 295–305, https://www.nature.com/articles/s41416‐019‐0603‐6. 10.1038/s41416-019-0603-6 PMC7000822 31666668

[58] W. G. Breen , M. P. Aryal , Y. Cao , and M. M. Kim , “Integrating Multi‐modal Imaging in Radiation Treatments for Glioblastoma,” Neuro‐Oncology 26, no. 12 (2024): S17–S25, 10.1093/neuonc/noad187. PMC10911793 38437666

[59] P. Grossmann , V. Narayan , K. Chang , et al., “Quantitative Imaging Biomarkers for Risk Stratification of Patients With Recurrent Glioblastoma Treated With bevacizumab,” Neuro‐Oncology 19, no. 12 (2017): 1688–1697, 10.1093/neuonc/nox092. PMC5716072 28499022

[60] M. Ratliff , H. Kim , H. Qi , et al., “Patient‐Derived Tumor Organoids for Guidance of Personalized Drug Therapies in Recurrent Glioblastoma,” International Journal of Molecular Sciences 23, no. 12 (2022): 6572, 10.3390/ijms23126572. PMC9223608 35743016

[61] S. Kudruk , C. M. Forsyth , M. Z. Dion , et al., “Multimodal Neuro‐nanotechnology: Challenging the Existing Paradigm in Glioblastoma Therapy,” Proceedings of the National Academy of Sciences 121, no. 8 (2024): 2306973121, 10.1073/pnas.2306973121. PMC10895370 38346200

[62] “Surgical Neurology International.” Accessed: May 04, 2026. [Online]. Available: https://surgicalneurologyint.com/surgicalint‐articles/ethical‐and‐therapeutic‐dilemmas‐in‐glioblastoma‐management‐during‐pregnancy‐two‐case‐reports‐and‐review‐of‐the‐literature/.

[63] S. Yang , H. Hu , H. Kung , et al., “Organoids: The Current Status and Biomedical Applications,” MedComm 4, no. 3 (2023): 274, 10.1002/mco2.274. PMC10192887 37215622

[64] G. Sean , A. J. Banes , and R. Gangaraju , “Organoids and Tissue/Organ Chips,” Stem Cell Research & Therapy 15 (2024): 241, https://link.springer.com/article/10.1186/s13287‐024‐03859‐1. 10.1186/s13287-024-03859-1 PMC11299405 39098898

[65] S. Bergmann , S. E. Lawler , Y. Qu , et al., “Blood–brain‐barrier Organoids for Investigating the Permeability of CNS Therapeutics,” Nature Protocols 13, no. 12 (2018): 2827–2843, 10.1038/s41596-018-0066-x. PMC6673652 30382243

[66] C. G. Hubert , M. Rivera , L. C. Spangler , et al., “A Three‐Dimensional Organoid Culture System Derived From Human Glioblastomas Recapitulates the Hypoxic Gradients and Cancer Stem Cell Heterogeneity of Tumors Found in Vivo,” Cancer Research 76 (2016): 2465–2477, https://aacrjournals.org/cancerres/article/76/8/2465/617390/A‐Three‐Dimensional‐Organoid‐Culture‐System. 10.1158/0008-5472.CAN-15-2402 PMC4873351 26896279

[67] C. Wang , M. Sun , C. Shao , et al., “A Multidimensional Atlas of human Glioblastoma‐Like Organoids Reveals Highly Coordinated Molecular Networks and Effective Drugs,” npj Precision Oncology 8, no. 1 (2024): 19, 10.1038/s41698-024-00500-5. PMC10811239 38273014

[68] S. Bian , M. Repic , Z. Guo , et al., “Genetically Engineered Cerebral Organoids Model Brain Tumor Formation,” Nature Methods 15, no. 8 (2018): 631–639, 10.1038/s41592-018-0070-7. PMC6071863 30038414

[69] M. T. Pham , K. M. Pollock , M. D. Rose , et al., “Generation of human Vascularized Brain Organoids,” Neuroreport 29, no. 7 (2018): 588–593, 10.1097/WNR.0000000000001014. PMC6476536 29570159

[70] E. Klein , A.‐C. Hau , A. Oudin , A. Golebiewska , and S. P. Niclou , “Glioblastoma Organoids: Pre‐Clinical Applications and Challenges in the Context of Immunotherapy,” Frontiers in Oncology 10 (2020): 604121, 10.3389/fonc.2020.604121. PMC7753120 33364198

[71] J. Wang , X. Tao , J. Zhu , et al., “Tumor Organoid‐Immune Co‐Culture Models: Exploring a New Perspective of Tumor Immunity,” Cell Death Discovery 11 (2025): 195, https://www.nature.com/articles/s41420‐025‐02407‐x. 10.1038/s41420-025-02407-x PMC12019369 40268893

[72] F. Watanabe , E. W. Hollingsworth , J. M. Bartley , et al., “Patient‐derived Organoids Recapitulate Glioma‐intrinsic Immune Program and Progenitor Populations of Glioblastoma,” PNAS Nexus 3, no. 2 (2024): pgae051, 10.1093/pnasnexus/pgae051. PMC10879747 38384384

[73] K. D. Pawlowski , J. T. Duffy , M. V. Babak , and I. V. Balyasnikova , “Modeling Glioblastoma Complexity With Organoids for Personalized Treatments,” Trends in Molecular Medicine 29 (2023): p282–296, https://www.cell.com/trends/molecular‐medicine/fulltext/S1471‐4914(23)00016‐3?rss=yes. 10.1016/j.molmed.2023.01.002 PMC11101135 36805210

[74] M. Logun , X. Wang , Y. Sun , et al., “Patient‐derived Glioblastoma Organoids as Real‐time Avatars for Assessing Responses to Clinical CAR‐T Cell Therapy,” Cell Stem Cell 32, no. 2 (2025): 181–190.e4, 10.1016/j.stem.2024.11.010. PMC11808387 39657679

[75] A. S. Tatla , A. W. Justin , C. Watts , and A. E. Markaki , “A Vascularized Tumoroid Model for human Glioblastoma Angiogenesis,” Scientific Reports 11 (2021): 19550, https://www.nature.com/articles/s41598‐021‐98911‐y. 10.1038/s41598-021-98911-y PMC8486855 34599235

[76] G. Pinto , I. Saenz‐de‐Santa‐Maria , P. Chastagner , et al., “Patient‐derived Glioblastoma Stem Cells Transfer Mitochondria Through Tunneling Nanotubes in Tumor Organoids,” Biochemical Journal 478 (2021): 21–29, https://portlandpress.com/biochemj/article/478/1/21/227071/Patient‐derived‐glioblastoma‐stem‐cells‐transfer. 10.1042/BCJ20200710 PMC7800365 33245115

[77] B. C. Prager , S. Bhargava , V. Mahadev , C. G. Hubert , and J. N. Rich , “Glioblastoma Stem Cells: Driving Resilience Through Chaos,” Trends in Cancer 6, no. 3 (2020): 223–235, 10.1016/j.trecan.2020.01.009. PMC8779821 32101725

[78] A. Golebiewska , A.‐C. Hau , A. Oudin , et al., “Patient‐derived Organoids and Orthotopic Xenografts of Primary and Recurrent Gliomas Represent Relevant Patient Avatars for Precision Oncology,” Acta Neuropathologica 140, no. 6 (2020): 919–949, 10.1007/s00401-020-02226-7. PMC7666297 33009951

[79] A. Wöllner , A. Paul , M. Arquilla , et al., “Use of Tissue Specimens From Stereotactic Biopsies for Patient‐Derived GBM Organoid‐Based Drug Testing,” Cells 14, no. 10 (2025): 701, 10.3390/cells14100701. PMC12109714 40422204

[80] M. Maruyama , Y. Nakano , T. Nishimura , et al., “PC3‐Secreted Microprotein Is Expressed in Glioblastoma Stem‐Like Cells and Human Glioma Tissues,” Biological and Pharmaceutical Bulletin 44, no. 7 (2021): 910–919, 10.1248/bpb.b20-00868. 33896885

[81] J. D. Lathia , S. C. Mack , E. E. Mulkearns‐Hubert , C. L. L. Valentim , and J. N. Rich , “Cancer Stem Cells in Glioblastoma,” Genes and Development 29 (2015): 1203–1217, https://genesdev.cshlp.org/content/29/12/1203. 10.1101/gad.261982.115 PMC4495393 26109046

[82] S. K. Singh , C. Hawkins , I. D. Clarke , et al., “Identification of human Brain Tumour Initiating Cells,” Nature 432 (2004): 396–401, https://www.nature.com/articles/nature03128. 10.1038/nature03128 15549107

[83] K. A. Schalper , M. E. Rodriguez‐Ruiz , R. Diez‐Valle , et al., “Neoadjuvant Nivolumab Modifies the Tumor Immune Microenvironment in Resectable Glioblastoma,” Nature Medicine 25 (2019): 470–476, https://www.nature.com/articles/s41591‐018‐0339‐5. 10.1038/s41591-018-0339-5 30742120

[84] Z. Zhao , X. Chen , A. M. Dowbaj , et al., “Organoids,” Nature Reviews Methods Primers 2 (2022): 94, https://www.nature.com/articles/s43586‐022‐00174‐y. 10.1038/s43586-022-00174-y PMC10270325 37325195

[85] H. Tiriac , J. C. Bucobo , D. Tzimas , et al., “Successful Creation of Pancreatic Cancer Organoids by Means of EUS‐guided Fine‐needle Biopsy Sampling for Personalized Cancer Treatment,” Gastrointestinal Endoscopy 87, no. 6 (2018): 1474–1480, 10.1016/j.gie.2017.12.032. PMC6143289 29325707

[86] J. Kim , B.‐K. Koo , and J. A. Knoblich , “Human Organoids: Model Systems for human Biology and Medicine,” Nature Reviews Molecular Cell Biology 21, no. 10 (2020): 571–584, 10.1038/s41580-020-0259-3. PMC7339799 32636524

[87] J. Ogawa , G. M. Pao , M. N. Shokhirev , and I. M. Verma , “Glioblastoma Model Using Human Cerebral Organoids,” Cell Reports 23, no. 4 (2018): 1220–1229, 10.1016/j.celrep.2018.03.105. PMC6892608 29694897

[88] R. Azzarelli , “Organoid Models of Glioblastoma to Study Brain Tumor Stem Cells,” Frontiers in Cell and Developmental Biology 8 (2020): 00220, 10.3389/fcell.2020.00220. PMC7176979 32373607

[89] I. Teriyapirom , A. S. Batista‐Rocha , and B.‐K. Koo , “Genetic Engineering in Organoids,” Journal of Molecular Medicine 99, no. 4 (2021): 555–568, 10.1007/s00109-020-02029-z. PMC8026415 33459801

[90] N. K. Karamanos , A. D. Theocharis , Z. Piperigkou , et al., “A Guide to the Composition and Functions of the Extracellular Matrix,” Karamanos 288 (2021): 6850–6912, https://febs.onlinelibrary.wiley.com/doi/10.1111/febs.15776. 10.1111/febs.15776 33605520

[91] Y. Ni , X. Zhou , J. Yang , H. Shi , H. Li , X. Zhao , and X. Ma , “The Role of Tumor‐Stroma Interactions in Drug Resistance within Tumor Microenvironment,” Frontiers in Cell and Developmental Biology 9 (2021): 637675, 10.3389/fcell.2021.637675. PMC8173135 34095111

[92] J. H. Heo , D. Kang , S. J. Seo , and Y. Jin , “Engineering the Extracellular Matrix for Organoid Culture,” International Journal of Stem Cells 15, no. 1 (2022): 60–69, 10.15283/ijsc21190. PMC8889330 35220292

[93] L. Sun , Y. Jiang , H. Tan , and R. Liang , “Collagen and Derivatives‐based Materials as Substrates for the Establishment of Glioblastoma Organoids,” International Journal of Biological Macromolecules 254 (2024): 128018, 10.1016/j.ijbiomac.2023.128018. 37967599

[94] C. Niu , C. J. Fisher , K. Scheffler , et al., “Polyacrylamide Gel Substrates That Simulate the Mechanical Stiffness of Normal and Malignant Neuronal Tissues Increase Protoporphyin IX Synthesis in Glioma Cells,” Journal of Biomedical Optics 20 (2015): 098002. 10.1117/1.JBO.20.9.098002 26405823

[95] Z. Yin , A. J. Romano , A. Manduca , R. L. Ehman , and J. I. Huston , “Stiffness and beyond: What MR Elastography Can Tell Us about Brain Structure and Function under Physiologic and Pathologic Conditions,” Topics in Magnetic Resonance Imaging 27, no. 5 (2018): 305, 10.1097/RMR.0000000000000178. PMC6176744 30289827

[96] E. M. Carvalho , E. A. Ding , A. Saha , et al., “Viscoelastic High‐molecular‐weight Hyaluronic Acid Hydrogels Support Rapid Glioblastoma Cell Invasion With Leader‐follower Dynamics,” BioRxiv 36 (2024): 2404885, 10.1101/2024.04.04.588167. PMC11637900 39508297

[97] L. Oldak , S. Chludzinska‐Kasperuk , P. Milewska , K. Grubczak , J. Reszec , and E. Gorodkiewicz , “Laminin‐5, Fibronectin, and Type IV Collagen as Potential Biomarkers of Brain Glioma Malignancy,” Biomedicines 10, no. 9 (2022): 2290, 10.3390/biomedicines10092290. PMC9496479 36140391

[98] T. Stankovic , T. Randelovic , M. Dragoj , et al., “ *In Vitro* Biomimetic Models for Glioblastoma‐a Promising Tool for Drug Response Studies,” Drug Resistance Updates 55 (2021): 100753, 10.1016/j.drup.2021.100753. 33667959

[99] E. Begagic , H. Beculic , A. Dzidic‐Krivic , et al., “Understanding the Significance of Hypoxia‐Inducible Factors (HIFs) in Glioblastoma: A Systematic Review,” Cancers 16, no. 11 (2024): 2089, 10.3390/cancers16112089. PMC11171068 38893207

[100] F. K. Braun , T. Rothhammer‐Hampl , J. Lorenz , et al., “Scaffold‐Based (Matrigel™) 3D Culture Technique of Glioblastoma Recovers a Patient‐Like Immunosuppressive Phenotype,” Cells 12, no. 14 (2023): 1856, 10.3390/cells12141856. PMC10378658 37508520

[101] H. Song , T. Guo , Z. Zhao , et al., “Biocompatible PEGylated Gold Nanorods Function as Cytokinesis Inhibitors to Suppress Angiogenesis,” Biomaterials 178 (2018): 23–35, 10.1016/j.biomaterials.2018.06.006. 29908342

[102] N. Bayat , R. Izadpanah , S. Ebrahimi‐Barough , et al., “The Anti‐Angiogenic Effect of Atorvastatin in Glioblastoma Spheroids Tumor Cultured in Fibrin Gel: In 3D in Vitro Model,” Asian Pacific journal of cancer prevention: APJCP 19, no. 9 (2018): 2553–2560, 10.22034/APJCP.2018.19.9.2553. PMC6249458 30256055

[103] P. P. Partyka , G. A. Godsey , J. R. Galie , et al., “Mechanical Stress Regulates Transport in a Compliant 3D Model of the Blood‐brain Barrier,” Biomaterials 115 (2017): 30–39, 10.1016/j.biomaterials.2016.11.012. 27886553

[104] E. A. Aisenbrey and W. L. Murphy , “Synthetic Alternatives to Matrigel,” Nature Reviews Materials 5, no. 7 (2020): 539–551, 10.1038/s41578-020-0199-8. PMC7500703 32953138

[105] A. Sedlář , M. Trávníčková , R. Matějka , et al., “Growth Factors VEGF‐A165 and FGF‐2 as Multifunctional Biomolecules Governing Cell Adhesion and Proliferation,” International Journal of Molecular Sciences 22, no. 4 (2021): 1843, 10.3390/ijms22041843. PMC7917819 33673317

[106] K. D. Pawlowski , J. T. Duffy , M. V. Babak , and I. V. Balyasnikova , “Modeling Glioblastoma Complexity With Organoids for Personalized Treatments,” Trends in Molecular Medicine 29, no. 4 (2023): 282–296, 10.1016/j.molmed.2023.01.002. PMC11101135 36805210

[107] J. Wen , F. Liu , Q. Cheng , et al., “Applications of Organoid Technology to Brain Tumors,” CNS Neuroscience & Therapeutics 29 (2023): 2725–2743, https://onlinelibrary.wiley.com/doi/10.1111/cns.14272. 10.1111/cns.14272 PMC10493676 37248629

[108] X. Wang , Y. Sun , D. Y. Zhang , G. Ming , and H. Song , “Glioblastoma Modeling With 3D Organoids: Progress and Challenges,” Oxford Open Neuroscience 2 (2023): kvad008, 10.1093/oons/kvad008. PMC10913843 38596241

[109] X.‐Y. Tang , S. Wu , D. Wang , et al., “Human Organoids in Basic Research and Clinical Applications,” Signal Transduction and Targeted Therapy 7, no. 1 (2022): 168, 10.1038/s41392-022-01024-9. PMC9127490 35610212

[110] K. Valyi‐Nagy , F. Betsou , A. Susma , and T. Valyi‐Nagy , “Optimization of Viable Glioblastoma Cryopreservation for Establishment of Primary Tumor Cell Cultures,” Biopreservation and Biobanking 19, no. 1 (2021): 60–66, 10.1089/bio.2020.0050. PMC7892309 33107762

[111] O. Rogulska , J. Havelkova , and Y. Petrenko , “Cryopreservation of Organoids,” Cryoletters 44, no. 2 (2023): 65–75, 10.54680/fr23210110112. 37883156

[112] M. Mendy , E. Caboux , R. T. Lawlor , J. Wright , and C. P. Wild , “Role of Biobanks in Cancer research,” in Common Minimum Technical Standards and Protocols for Biobanks Dedicated to Cancer Research, (International Agency for Research on Cancer, 2017), https://www.ncbi.nlm.nih.gov/books/NBK567260/. 33539055

[113] E. Darrigues , E. H. Zhao , A. De Loose , et al., “Biobanked Glioblastoma Patient‐Derived Organoids as a Precision Medicine Model to Study Inhibition of Invasion,” International Journal of Molecular Sciences 22, no. 19 (2021): 10720, 10.3390/ijms221910720. PMC8509225 34639060

[114] A. Linkous , D. Balamatsias , M. Snuderl , et al., “Modeling Patient‐Derived Glioblastoma With Cerebral Organoids,” Cell Reports 26, no. 12 (2019): 3203–3211.e5, 10.1016/j.celrep.2019.02.063. PMC6625753 30893594

[115] T. G. Krieger , S. M. Tirier , J. Park , et al., “Modeling Glioblastoma Invasion Using human Brain Organoids and Single‐cell Transcriptomics,” Neuro‐Oncology 22, no. 8 (2020): 1138–1149, 10.1093/neuonc/noaa091. PMC7594554 32297954

[116] P. Zhuang , B. Scott , S. Gao , et al., “Blood‐tumor Barrier Organoids Recapitulate Glioblastoma Microenvironment and Enable High‐throughput Modeling of Therapeutic Delivery,” BioRxiv (2025): 622979, 10.1101/2024.11.11.622979.

[117] F. Jacob , G. Ming , and H. Song , “Generation and Biobanking of Patient‐derived Glioblastoma Organoids and Their Application in CAR T Cell Testing,” Nature Protocols 15 (2020): 4000–4033, https://www.nature.com/articles/s41596‐020‐0402‐9. 10.1038/s41596-020-0402-9 33169003

[118] S. El Harane , B. Zidi , N. El Harane , K.‐H. Krause , T. Matthes , and O. Preynat‐Seauve , “Cancer Spheroids and Organoids as Novel Tools for Research and Therapy: State of the Art and Challenges to Guide Precision Medicine,” Cells 12, no. 7 (2023): 1001, 10.3390/cells12071001. PMC10093533 37048073

[119] N. Silvia and G. Dai , “Cerebral Organoids as a Model for Glioblastoma Multiforme,” Current Opinion in Biomedical Engineering 13 (2020): 152–159, 10.1016/j.cobme.2020.03.004. PMC7192544 32355905

[120] F. R. Weth , L. Peng , E. Paterson , S. T. Tan , and C. Gray , “Utility of the Cerebral Organoid Glioma ‘GLICO’ Model for Screening Applications,” Cells 12, no. 1 (2022): 153, 10.3390/cells12010153. PMC9818141 36611949

[121] H. A. Fine , “GLICO. Platform: Patient‐Specific Brain Tumor Organoids for Precision Drug Development,” Enterprise Innovation https://innovation.weill.cornell.edu/industry‐investors‐partners/technology‐portfolio/glico‐platform‐patient‐specific‐brain‐tumor, Accessed: May 06, 2026. [Online]. Available.

[122] logosbio , “3D Cell Culture: Differences between Spheroids and Organoids and Their Analytical Strategies,” *Logos Biosystems Accessed*: 2026. [Online]. Available: https://logosbio.com/3d‐cell‐culture‐differences‐between‐spheroids‐and‐organoids‐and‐their‐analytical‐strategies/.

[123] “Spheroids Organoids: The Difference Between The 3D Cultures *Bio‐Techne*” Accessed: May 06, 2026. [Online]. Available: https://www.bio‐techne.com/resources/blogs/spheroids‐vs‐organoids‐which‐3d‐cell‐culture‐model‐is‐best.

[124] J. Lim , H. Ching , J.‐K. Yoon , N. L. Jeon , and Y. Kim , “Microvascularized Tumor Organoids‐on‐chips: Advancing Preclinical Drug Screening With Pathophysiological Relevance,” Nano Convergence 8, no. 1 (2021): 12, 10.1186/s40580-021-00261-y. PMC8042002 33846849

[125] A. Mariappan , G. Goranci‐Buzhala , L. Ricci‐Vitiani , R. Pallini , and J. Gopalakrishnan , “Trends and Challenges in Modeling Glioma Using 3D human Brain Organoids,” Cell Death & Differentiation 28 (2021): 15–23. 10.1038/s41418-020-00679-7 PMC7707134 33262470

[126] Y.‐W. Yan , E. S. Qian , L. E. Woodard , and J. Bejoy , “Neural Lineage Differentiation of human Pluripotent Stem Cells: Advances in Disease Modeling,” World Journal of Stem Cells 15, no. 6 (2023): 530–547, 10.4252/wjsc.v15.i6.530. PMC10324500 37424945

[127] L. A. Mulder , J. A. Depla , A. Sridhar , K. Wolthers , D. Pajkrt , and R. Vieira de Sá , “A Beginner's Guide on the Use of Brain Organoids for Neuroscientists: A Systematic Review,” Stem Cell Research & Therapy 14 (2023): 87, 10.1186/s13287-023-03302-x. PMC10105545 37061699

[128] R. Lugano , M. Ramachandran , and A. Dimberg , “Tumor Angiogenesis: Causes, Consequences, Challenges and Opportunities,” Cellular and Molecular Life Sciences 77 (2020): 1745–1770, https://link.springer.com/article/10.1007/s00018‐019‐03351‐7. 10.1007/s00018-019-03351-7 PMC7190605 31690961

[129] P. Wörsdörfer , N. Dalda , A. Kern , et al., “Generation of Complex human Organoid Models Including Vascular Networks by Incorporation of Mesodermal Progenitor Cells,” Scientific Reports 9 (2019): 15663, https://www.nature.com/articles/s41598‐019‐52204‐7. 10.1038/s41598-019-52204-7 PMC6821804 31666641

[130] W. Duan , S. Xia , M. Tang , M. Lin , W. Liu , and Q. Wang , “Targeting of Endothelial Cells in Brain Tumours,” Clinical and Translational Medicine 13, no. 10 (2023): 1433, 10.1002/ctm2.1433. PMC10570772 37830128

[131] S. Zhang , Z. Wan , and R. D. Kamm , “Vascularized Organoids on a Chip: Strategies for Engineering Organoids With Functional Vasculature,” Lab on a Chip 21, no. 3 (Feb. 2021): 473–488, 10.1039/D0LC01186J. PMC8283929 33480945

[132] S. R. Moses , J. J. Adorno , A. F. Palmer , and J. W. Song , “Vessel‐on‐a‐chip Models for Studying Microvascular Physiology, Transport, and Function in Vitro,” American Journal of Physiology‐Cell Physiology 320, no. 1 (2021): C92–C105, 10.1152/ajpcell.00355.2020. PMC7846973 33176110

[133] G. Hovis , N. Chandra , N. Kejriwal , et al., “Understanding the Role of Endothelial Cells in Glioblastoma,” Mechanisms and Novel Treatments 25 (2024): 6118, https://www.mdpi.com/1422‐0067/25/11/6118. 10.3390/ijms25116118 PMC11173095 38892305

[134] K. M. Noh , S.‐J. Park , S.‐H. Moon , and S. Y. Jung , “Extracellular Matrix Cues Regulate the Differentiation of Pluripotent Stem Cell‐derived Endothelial Cells,” Frontiers in Cardiovascular Medicine 10 (2023): 1169331, 10.3389/fcvm.2023.1169331. PMC10330705 37435057

[135] T. Russell , Q. Dirar , Y. Li , et al., “Cortical Spheroid on Perfusable Microvascular Network in a Microfluidic Device,” PLOS One (2023): 0288025, https://journals.plos.org/plosone/article?id=10.1371/journal.pone.0288025. 10.1371/journal.pone.0288025 PMC10586606 37856438

[136] M. G. Kook , S.‐E. Lee , N. Shin , et al., “Generation of Cortical Brain Organoid With Vascularization by Assembling With Vascular Spheroid,” International Journal of Stem Cells 15, no. 1 (2022): 85–94, 10.15283/ijsc21157. PMC8889335 35220294

[137] S. Y. Tan , X. Feng , L. K. W. Cheng , and A. R. Wu , “Vascularized human Brain Organoid on‐chip,” Lab on a Chip 23, no. 12 (2023): 2693–2709, 10.1039/D2LC01109C. 37256563

[138] R. Simsa , T. Rothenbücher , H. Gürbüz , et al., “Brain Organoid Formation on Decellularized Porcine Brain ECM Hydrogels,” PLoS ONE 16, no. 1 (2021): 0245685, 10.1371/journal.pone.0245685. PMC7842896 33507989

[139] W. Xue , X. Du , H. Wu , et al., “Aberrant Glioblastoma Neovascularization Patterns and Their Correlation With DCE‐MRI‐derived Parameters Following Temozolomide and Bevacizumab Treatment,” Scientific Reports 7, no. 1 2017): 13894, 10.1038/s41598-017-14341-9. PMC5654943 29066764

[140] R. J. Diaz , S. Ali , M. G. Qadir , M. I. De La Fuente , M. E. Ivan , and R. J. Komotar , “The Role of Bevacizumab in the Treatment of Glioblastoma,” Journal of Neuro‐Oncology 133 (2017): 455–467, https://link.springer.com/article/10.1007/s11060‐017‐2477‐x. 10.1007/s11060-017-2477-x 28527008

[141] S. Rosińska and J. Gavard , “Tumor Vessels Fuel the Fire in Glioblastoma,” International Journal of Molecular Sciences 22, no. 12 (2021): 6514, 10.3390/ijms22126514. PMC8235363 34204510

[142] T. E. Peterson , N. D. Kirkpatrick , Y. Huang , et al., “Dual Inhibition of Ang‐2 and VEGF Receptors Normalizes Tumor Vasculature and Prolongs Survival in Glioblastoma by Altering Macrophages,” PNAS 113 (2016): 4470–4475, https://www.pnas.org/doi/full/10.1073/pnas.1525349113. 10.1073/pnas.1525349113 PMC4843449 27044097

[143] S. Conroy , F. A. E. Kruyt , M. Wagemakers , K. P. L. Bhat , and W. F. A. den Dunnen , “IL‐8 associates With a pro‐angiogenic and mesenchymal subtype in glioblastoma,” Oncotarget 9, no. 21 (2018): 15721–15731, 10.18632/oncotarget.24595. PMC5884659 29644004

[144] F. Daneshimehr , Z. Barabadi , S. Abdolahi , et al., “Angiogenesis and Its Targeting in Glioblastoma With Focus on Clinical Approaches,” Cell Journal (Yakhteh) 24, no. 10 (2022): 555–568, 10.22074/cellj.2022.8154. PMC9617020 36259473

[145] M. E. Hardee and D. Zagzag , “Mechanisms of Glioma‐Associated Neovascularization,” The American Journal of Pathology 181 (2012): 1126–1141, https://ajp.amjpathol.org/article/S0002‐9440(12)00508‐1/fulltext. 10.1016/j.ajpath.2012.06.030 PMC3463636 22858156

[146] C. A. Shaifer , J. Huang , and P. C. Lin , “Glioblastoma Cells Incorporate Into Tumor Vasculature and Contribute to Vascular Radioresistance,” International Journal of Cancer 127 (2010): 2063–2075, https://onlinelibrary.wiley.com/doi/10.1002/ijc.25249. 10.1002/ijc.25249 PMC2932815 20162571

[147] X. Liu , Q. Zhang , Y. Mu , et al., “Clinical Significance of Vasculogenic Mimicry in human Gliomas,” Journal of Neuro‐Oncology 105 (2011): 173–179, https://link.springer.com/article/10.1007/s11060‐011‐0578‐5. 10.1007/s11060-011-0578-5 PMC3198193 21533525

[148] K. Maddison , N. A. Bowden , M. C. Graves , and P. A. Tooney , “Characteristics of Vasculogenic Mimicry and Tumour to Endothelial Transdifferentiation in human Glioblastoma: A Systematic Review,” BMC cancer 23, no. 1 (2023): 185, 10.1186/s12885-023-10659-y. PMC9948311 36823554

[149] S. Baisiwala , B. Auffinger , S. P. Caragher , et al., “Chemotherapeutic Stress Induces Transdifferentiation of Glioblastoma Cells to Endothelial Cells and Promotes Vascular Mimicry,” Stem Cells International (2019): 6107456, https://onlinelibrary.wiley.com/doi/10.1155/2019/6107456. 10.1155/2019/6107456 PMC6604352 31316566

[150] P. Deshors , C. Toulas , F. Arnauduc , et al., “Ionizing Radiation Induces Endothelial Transdifferentiation of Glioblastoma Stem‐Like Cells Through the Tie2 Signaling Pathway,” Cell Death & Disease 10 (2019): 816, https://www.nature.com/articles/s41419‐019‐2055‐6. 10.1038/s41419-019-2055-6 PMC6817826 31659157

[151] J. C. Carlson , M. C. Gutierrez , B. Lozzi , et al., “Identification of Diverse Tumor Endothelial Cell Populations in Malignant Glioma,” Neuro‐Oncology Oxford Academic 23 (2021): 932–944, https://academic.oup.com/neuro‐oncology/article/23/6/932/6046199. 10.1093/neuonc/noaa297 PMC8168823 33367832

[152] C. Zhao , G. A. Gomez , Y. Zhao , et al., “ETV2 mediates Endothelial Transdifferentiation of Glioblastoma,” Signal Transduction and Targeted Therapy 3 (2018): 4, https://www.nature.com/articles/s41392‐018‐0007‐8. 10.1038/s41392-018-0007-8 PMC5837107 29527330

[153] Y. Soda , C. Myskiw , A. Rommel , and I. M. Verma , “Mechanisms of Neovascularization and Resistance to Anti‐angiogenic Therapies in Glioblastoma Multiforme,” Journal of Molecular Medicine 91 (2013): 439–448, https://link.springer.com/article/10.1007/s00109‐013‐1019‐z. 10.1007/s00109-013-1019-z PMC3665343 23512266

[154] A. Chandra , A. Jahangiri , W. Chen , et al., “Clonal ZEB1‐Driven Mesenchymal Transition Promotes Targetable Oncologic Antiangiogenic Therapy Resistance,” Cancer Research 80 (2020): 1498–1511, https://aacrjournals.org/cancerres/article/80/7/1498/647742/Clonal‐ZEB1‐Driven‐Mesenchymal‐Transition‐Promotes. 10.1158/0008-5472.CAN-19-1305 PMC7236890 32041837

[155] J. Bae , M.‐H. Kim , S. Han , and S. Park , “Development of Tumor‐Vasculature Interaction on Chip Mimicking Vessel Co‐Option of Glioblastoma,” BioChip Journal 17 (2023): 77–84, https://link.springer.com/article/10.1007/s13206‐022‐00090‐z.

[156] Y. Wang , F. Zhang , N. Xiong , et al., “Remodelling and Treatment of the Blood‐Brain Barrier in Glioma,” CMAR 13 (2021): 4217–4232, 10.2147/CMAR.S288720. PMC8166259 34079374

[157] F. Torrisi , C. Alberghina , S. D'Aprile , et al., “The Hallmarks of Glioblastoma: Heterogeneity, Intercellular Crosstalk and Molecular Signature of Invasiveness and Progression,” Biomedicines 10, no. 4 (2022): 806, 10.3390/biomedicines10040806. PMC9031586 35453557

[158] M. Buccarelli , G. Castellani , and L. Ricci‐Vitiani , “Glioblastoma‐Specific Strategies of Vascularization: Implications in Anti‐Angiogenic Therapy Resistance,” Journal of Personalized Medicine 12, no. 10 (2022): 1625, 10.3390/jpm12101625. PMC9604754 36294763

[159] D. Belotti , D. Pinessi , and G. Taraboletti , “Alternative Vascularization Mechanisms in Tumor Resistance to Therapy,” Cancers 13, no. 8 (2021): 1912, 10.3390/cancers13081912. PMC8071410 33921099

[160] E. A. Kuczynski and A. R. Reynolds , “Vessel co‐option and Resistance to Anti‐angiogenic Therapy,” Angiogenesis 23 (2020): 55–74, https://link.springer.com/article/10.1007/s10456‐019‐09698‐6. 10.1007/s10456-019-09698-6 31865479

[161] S. A. Liddelow , “Development of the Choroid Plexus and Blood‐CSF Barrier,” Frontiers in Neuroscience 9 (2015): 32, https://www.frontiersin.org/journals/neuroscience/articles/10.3389/fnins.2015.00032/full. 10.3389/fnins.2015.00032 PMC4347429 25784848

[162] P. Solár , A. Zamani , L. Kubíčková , P. Dubový , and M. Joukal , “Choroid Plexus and the Blood–cerebrospinal Fluid Barrier in Disease,” Fluids and Barriers of the CNS 17 (2020): 35, https://link.springer.com/article/10.1186/s12987‐020‐00196‐2. 10.1186/s12987-020-00196-2 PMC7201396 32375819

[163] J. Laterra , R. Keep , L. A. Betz , and G. W. Goldstein , “Blood—Cerebrospinal Fluid Barrier,” in Basic Neurochemistry: Molecular, Cellular and Medical Aspects , 6th edition (Lippincott‐Raven, 1999), https://www.ncbi.nlm.nih.gov/books/NBK27998/.

[164] D. Verhaege , C. De Nolf , L. Van Acker , et al., “Base Barrier Cells Provide Compartmentalization of Choroid Plexus, Brain and CSF,” Nature Neuroscience 29 (2026): 551–566, https://www.nature.com/articles/s41593‐025‐02188‐7. 10.1038/s41593-025-02188-7 PMC12971494 41680326

[165] P. Majerova , K. Khiratkar , K. James , D. Olesova , J. Vegh , and A. Kovac , “Development of Blood‐cerebrospinal Fluid Barrier Model Expressing Pharmaceutically Important Transporters,” bioRxiv (2024): 576616, 10.1101/2024.01.22.576616.

[166] A. Mosteiro , L. Pedrosa , A. Ferrés , D. Diao , À. Sierra , and J. J. González , “The Vascular Microenvironment in Glioblastoma: A Comprehensive Review,” Biomedicines 10, no. 6 (2022): 1285, 10.3390/biomedicines10061285. PMC9219822 35740307

[167] M. Peleli , A. Moustakas , and A. Papapetropoulos , “Endothelial‐Tumor Cell Interaction in Brain and CNS Malignancies,” International Journal of Molecular Sciences 21, no. 19 (2020): 7371, 10.3390/ijms21197371. PMC7582718 33036204

[168] M. Diksin , S. J. Smith , and R. Rahman , “The Molecular and Phenotypic Basis of the Glioma Invasive Perivascular Niche,” International Journal of Molecular Sciences 18, no. 11 (2017): 2342, 10.3390/ijms18112342. PMC5713311 29113105

[169] Y. T. Oh , H. J. Cho , J. Kim , et al., “Translational Validation of Personalized Treatment Strategy Based on Genetic Characteristics of Glioblastoma,” PLoS ONE 9, no. 8 (2014): 103327, 10.1371/journal.pone.0103327. PMC4118874 25084005

[170] M. Zou , Y. Duan , P. Wang , et al., “DYT‐40, a Novel Synthetic 2‐styryl‐5‐nitroimidazole Derivative, Blocks Malignant Glioblastoma Growth and Invasion by Inhibiting AEG‐1 and NF‐κB Signaling Pathways,” Scientific Reports 6 (2016): 27331, https://www.nature.com/articles/srep27331. 10.1038/srep27331 PMC4890319 27251589

[171] Z.‐B. Song , H.‐P. Yang , A.‐Q. Xu , et al., “Connective Tissue Growth Factor as an Unfavorable Prognostic Marker Promotes the Proliferation, Migration, and Invasion of Gliomas,” Chinese Medical Journal 133, no. 06 (2020): 670–678, 10.1097/CM9.0000000000000683. PMC7190229 32197031

[172] X. Guan , M. N. Hasan , S. Maniar , W. Jia , and D. Sun , “Reactive Astrocytes in Glioblastoma Multiforme,” Molecular Neurobiology 55, no. 8 (2018): 6927–6938, 10.1007/s12035-018-0880-8. 29363044

[173] S.‐J. Kim , H. J. Lee , M. S. Kim , et al., “Macitentan, a Dual Endothelin Receptor Antagonist, in Combination With Temozolomide Leads to Glioblastoma Regression and Long‐Term Survival in Mice,” Clinical Cancer Research 21, no. 20 (2015): 4630–4641, https://aacrjournals.org/clincancerres/article/21/20/4630/246203/Macitentan‐a‐Dual‐Endothelin‐Receptor‐Antagonist. 10.1158/1078-0432.CCR-14-3195 PMC4780217 26106074

[174] E. Henke , R. Nandigama , and S. Ergün , “Extracellular Matrix in the Tumor Microenvironment and Its Impact on Cancer Therapy,” Frontiers in Molecular Biosciences 6 (2020): 00160, 10.3389/fmolb.2019.00160. PMC7025524 32118030

[175] A. Belousov , S. Titov , N. Shved , et al., “The Extracellular Matrix and Biocompatible Materials in Glioblastoma Treatment,” Frontiers in Bioengineering and Biotechnology 7 (2019): 00341, 10.3389/fbioe.2019.00341. PMC6877546 31803736

[176] D. Kruglyakov , S. K. Ojha , M. Kartawy , et al., “Nitric Oxide Synthase Inhibition Prevents Cell Proliferation in Glioblastoma,” Journal of Molecular Neuroscience 73, no. 11 (2023): 875–883, 10.1007/s12031-023-02166-3. 37843719

[177] J. Chen , Y. Li , T.‐S. Yu , et al., “A Restricted Cell Population Propagates Glioblastoma Growth After Chemotherapy,” Nature 488 (2012): 522–526, https://www.nature.com/articles/nature11287. 10.1038/nature11287 PMC3427400 22854781

[178] A. Ishii , T. Kimura , H. Sadahiro , et al., “Histological Characterization of the Tumorigenic ‘Peri‐Necrotic Niche’ Harboring Quiescent Stem‐Like Tumor Cells in Glioblastoma,” PLOS One 11, no. 1 (2016): 0147366, https://journals.plos.org/plosone/article?id=10.1371/journal.pone.0147366. 10.1371/journal.pone.0147366 PMC4723051 26799577

[179] D. Schiffer , L. Annovazzi , C. Casalone , C. Corona , and M. Mellai , “Glioblastoma: Microenvironment and Niche Concept,” Cancers 11, no. 1 (2019): 5, 10.3390/cancers11010005. PMC6357107 30577488

[180] E. Jung , M. Osswald , M. Ratliff , et al., “Tumor Cell Plasticity, Heterogeneity, and Resistance in Crucial Microenvironmental Niches in Glioma,” Nature Communications 12 (2021): 1014, https://www.nature.com/articles/s41467‐021‐21117‐3. 10.1038/s41467-021-21117-3 PMC7881116 33579922

[181] M. T. Ngo , J. N. Sarkaria , and B. A. C. Harley , “Perivascular Stromal Cells Instruct Glioblastoma Invasion, Proliferation, and Therapeutic Response Within an Engineered Brain Perivascular Niche Model,” Advanced Science 9, no. 31 (2022): 2201888, 10.1002/advs.202201888. PMC9631060 36109186

[182] M. Gerigk , H. Bulstrode , H. H. Shi , et al., “On‐chip Perivascular Niche Supporting Stemness of Patient‐derived Glioma Cells in a Serum‐free, Flowable Culture,” Lab on a Chip 21, no. 12 (2021): 2343–2358, 10.1039/D1LC00271F. PMC8204159 33969368

[183] S. Kumar , L. Bar‐Lev , H. Sharife , et al., “Identification of Vascular Cues Contributing to Cancer Cell Stemness and Function,” Angiogenesis 25 (2022): 355–371, https://link.springer.com/article/10.1007/s10456‐022‐09830‐z. 10.1007/s10456-022-09830-z 35112158

[184] Y. Xiao , D. Kim , B. Dura , et al., “Ex Vivo Dynamics of Human Glioblastoma Cells in a Microvasculature‐on‐a‐Chip System Correlates With Tumor Heterogeneity and Subtypes,” Advanced Science 6 (2019): 1801531, https://advanced.onlinelibrary.wiley.com/doi/10.1002/advs.201801531. 10.1002/advs.201801531 PMC6468969 31016107

[185] R. Tejero , Y. Huang , I. Katsyv , et al., “Gene Signatures of Quiescent Glioblastoma Cells Reveal Mesenchymal Shift and Interactions With Niche Microenvironment,” EBioMedicine 42 (2019): 252–269, 10.1016/j.ebiom.2019.03.064. PMC6491796 30952620

[186] M. T. Ngo and B. A. C. Harley , “Perivascular Signals Alter Global Gene Expression Profile of Glioblastoma and Response to Temozolomide in a Gelatin Hydrogel,” Biomaterials 198 (2019): 122–134, 10.1016/j.biomaterials.2018.06.013. PMC6292767 29941152

[187] M. T. Ngo , E. Karvelis , and B. A. C. Harley , “Multidimensional Hydrogel Models Reveal Endothelial Network Angiocrine Signals Increase Glioblastoma Cell Number, Invasion, and Temozolomide Resistance,” Integrative Biology 12, no. 6 (2020): 139–149, 10.1093/intbio/zyaa010. PMC7296128 32507878

[188] S. Kumar , H. Sharife , T. Kreisel , et al., “Intra‐Tumoral Metabolic Zonation and Resultant Phenotypic Diversification Are Dictated by Blood Vessel Proximity,” Cell Metabolism 30, no. 1 (2019): 201–211.e6, 10.1016/j.cmet.2019.04.003. 31056286

[189] V. V. V. Hira , U. Verbovsek , B. Breznik , et al., “Cathepsin K Cleavage of SDF‐1α Inhibits Its Chemotactic Activity Towards Glioblastoma Stem‐Like Cells,” Biochimica et Biophysica Acta (BBA)—Molecular Cell Research 1864, no. 3 (2017): 594–603, 10.1016/j.bbamcr.2016.12.021. 28040478

[190] V. V. V. Hira , D. A. Aderetti , and C. J. F. van Noorden , “Glioma Stem Cell Niches in Human Glioblastoma Are Periarteriolar,” Journal of Histochemistry & Cytochemistry 66, no. 5 (2018): 349–358, 10.1369/0022155417752676. PMC5958355 29328867

[191] G. L. Gravina , A. Mancini , A. Colapietro , et al., “The Novel CXCR4 Antagonist, PRX177561, Reduces Tumor Cell Proliferation and Accelerates Cancer Stem Cell Differentiation in Glioblastoma Preclinical Models,” Tumor Biology 39, no. 6 (2017): 101042831769552, 10.1177/1010428317695528. 28639900

[192] N. H. Boyd , A. N. Tran , J. D. Bernstock , et al., “Glioma Stem Cells and Their Roles Within the Hypoxic Tumor Microenvironment,” Theranostics 11, no. 2 (2021): 665–683, 10.7150/thno.41692. PMC7738846 33391498

[193] C. A. A. Lee , P. Banerjee , B. J. Wilson , et al., “Targeting the ABC Transporter ABCB5 Sensitizes Glioblastoma to Temozolomide‐induced Apoptosis Through a Cell‐cycle Checkpoint Regulation Mechanism,” Journal of Biological Chemistry 295, no. 22 (2020): 7774–7788, 10.1074/jbc.RA120.013778. PMC7261782 32317280

[194] F. Wang , A. Y. Wang , C. Chesnelong , et al., “ING5 activity in Self‐renewal of Glioblastoma Stem Cells via Calcium and Follicle Stimulating Hormone Pathways,” Oncogene 37, no. 3 (2017): 286–301, https://www.nature.com/articles/onc2017324#Sec11. 10.1038/onc.2017.324 PMC5799773 28925404

[195] M. Messé , C. Bernhard , S. Foppolo , et al., “Hypoxia‐driven Heterogeneous Expression of α5 Integrin in Glioblastoma Stem Cells Is Linked to HIF‐2α,” Biochimica et Biophysica Acta (BBA)—Molecular Basis of Disease 1870, no. 8 (2024): 167471, 10.1016/j.bbadis.2024.167471. 39154793

[196] D. Maciaczyk , D. Picard , L. Zhao , et al., “CBF1 is Clinically Prognostic and Serves as a Target to Block Cellular Invasion and Chemoresistance of EMT‐Like Glioblastoma Cells,” British Journal of Cancer 117, no. 1 (2017): 102–112, 10.1038/bjc.2017.157. PMC5520214 28571041

[197] Y. Long , H. Tao , A. Karachi , et al., “Dysregulation of Glutamate Transport Enhances Treg Function That Promotes VEGF Blockade Resistance in Glioblastoma,” Cancer Research 80, no. 3 (2020): 499–509, 10.1158/0008-5472.CAN-19-1577. 31723000

[198] Y.‐T. Lin and K.‐J. Wu , “Epigenetic Regulation of Epithelial‐mesenchymal Transition: Focusing on Hypoxia and TGF‐β Signaling,” Journal of Biomedical Science 27 (2020): 39, https://link.springer.com/article/10.1186/s12929‐020‐00632‐3. 10.1186/s12929-020-00632-3 PMC7050137 32114978

[199] M. M. Barreca , C. Zichittella , R. Alessandro , and A. Conigliaro , “Hypoxia‐Induced Non‐Coding RNAs Controlling Cell Viability in Cancer,” International Journal of Molecular Sciences 22, no. 4 (2021): 1857, 10.3390/ijms22041857. PMC7918432 33673376

[200] G. Seano and R. K. Jain , “Vessel co‐option in Glioblastoma: Emerging Insights and Opportunities,” Angiogenesis 23 (2020): 9–16, https://link.springer.com/article/10.1007/s10456‐019‐09691‐z. 10.1007/s10456-019-09691-z PMC7012982 31679081

[201] F.‐C. Pai , H.‐W. Huang , Y.‐L. Tsai , et al., “Inhibition of FABP6 Reduces Tumor Cell Invasion and Angiogenesis Through the Decrease in MMP‐2 and VEGF in Human Glioblastoma Cells,” Cells 10, no. 10 (2021): 2782, 10.3390/cells10102782. PMC8534568 34685761

[202] A. Qin , A. Musket , P. R. Musich , J. B. Schweitzer , and Q. Xie , “Receptor Tyrosine Kinases as Druggable Targets in Glioblastoma: Do Signaling Pathways Matter?,” Neuro‐Oncology Advances 3, no. 1: vdab133, https://academic.oup.com/noa/article/3/1/vdab133/6371789. 10.1093/noajnl/vdab133 PMC8598918 34806012

[203] Y. L. Chen , Y. Luan , K. Ma , et al., “ISL1 Promotes Human Glioblastoma‐Derived Stem Cells′ Self‐Renewal by Activation of Sonic Hedgehog/GLI1 Function,” Stem Cells and Development 31 (2022): 0344, https://journals.sagepub.com/doi/abs/10.1089/scd.2021.0344. 10.1089/scd.2021.0344 35403434

[204] S. A. Richard , “The Pivotal Immunoregulatory Functions of Microglia and Macrophages in Glioma Pathogenesis and Therapy,” Journal of Oncology (2022): 8903482, 10.1155/2022/8903482. PMC9001141 35419058

[205] C. Barca , C. Foray , S. Hermann , et al., “The Colony Stimulating Factor‐1 Receptor (CSF‐1R)‐Mediated Regulation of Microglia/Macrophages as a Target for Neurological Disorders (Glioma, Stroke),” Frontiers in Immunology 12 (2021): 787307, 10.3389/fimmu.2021.787307. PMC8688767 34950148

[206] O. Cousins , A. Hodges , J. Schubert , et al., “The Blood–CSF–brain Route of Neurological Disease: The Indirect Pathway Into the Brain,” Neuropathology and Applied Neurobiology 48 (2022): 12789, https://onlinelibrary.wiley.com/doi/10.1111/nan.12789. 10.1111/nan.12789 34935179

[207] T. Shi , J. Zhu , X. Zhang , and X. Mao , “The Role of Hypoxia and Cancer Stem Cells in Development of Glioblastoma,” Cancers 15, no. 9 (2023): 2613, 10.3390/cancers15092613. PMC10177528 37174078

[208] S. Dallavalasa , N. M. Beeraka , C. G. Basavaraju , et al., “The Role of Tumor Associated Macrophages (TAMs) in Cancer Progression, Chemoresistance, Angiogenesis and Metastasis—Current Status,” Current Medicinal Chemistry 28, no. 39 (2021): 8203–8236, 10.2174/0929867328666210720143721. 34303328

[209] B. Kaminska and S. Cyranowski , “Recent Advances in Understanding Mechanisms of TGF Beta Signaling and Its Role in Glioma Pathogenesis,” in Glioma Signaling, ed. J. Barańska , (Springer International Publishing, 2020), 179–201, 10.1007/978-3-030-30651-9_9. 32034714

[210] X. Liu , Y. Liu , Y. Qi , et al., “Signal Pathways Involved in the Interaction between Tumor‐Associated Macrophages/TAMs and Glioblastoma Cells,” Frontiers in oncology 12 (2022): 822085, 10.3389/fonc.2022.822085. PMC9114701 35600367

[211] S. L. N. Maas , E. R. Abels , L. L. Van De Haar , et al., “Glioblastoma Hijacks Microglial Gene Expression to Support Tumor Growth,” Journal of Neuroinflammation 17, no. 1 (2020): 120, 10.1186/s12974-020-01797-2. PMC7164149 32299465

[212] K. Vinnakota , F. Hu , M.‐C. Ku , et al., “Toll‐Like Receptor 2 Mediates Microglia/Brain Macrophage MT1‐MMP Expression and Glioma Expansion,” Neuro‐Oncology 15, no. 11 (2013): 1457–1468, 10.1093/neuonc/not115. PMC3813418 24014382

[213] T. Miyazaki , E. Ishikawa , M. Matsuda , et al., “Infiltration of CD163‐positive Macrophages in Glioma Tissues After Treatment With Anti‐PD‐L1 Antibody and Role of PI3Kγ Inhibitor as a Combination Therapy With Anti‐PD‐L1 Antibody in in Vivo Model Using Temozolomide‐resistant Murine Glioma‐initiating Cells,” Brain Tumor Pathology 37, no. 2 (2020): 41–49, 10.1007/s10014-020-00357-z. 31980975

[214] L. Akkari , R. L. Bowman , J. Tessier , et al., “Dynamic Changes in Glioma Macrophage Populations After Radiotherapy Reveal CSF‐1R Inhibition as a Strategy to Overcome Resistance,” Science Translational Medicine 12, no. 552 (2020): aaw7843, 10.1126/scitranslmed.aaw7843. 32669424

[215] H.‐Y. Chuang , Y.‐K. Su , H.‐W. Liu , et al., “Preclinical Evidence of STAT3 Inhibitor Pacritinib Overcoming Temozolomide Resistance via Downregulating miR‐21‐Enriched Exosomes From M2 Glioblastoma‐Associated Macrophages,” Journal of Clinical Medicine 8, no. 7 (2019): 959, 10.3390/jcm8070959. PMC6678764 31269723

[216] E. F. Simonds , E. D. Lu , O. Badillo , et al., “Deep Immune Profiling Reveals Targetable Mechanisms of Immune Evasion in Immune Checkpoint Inhibitor‐refractory Glioblastoma,” Journal for ImmunoTherapy of Cancer 9, no. 6 (2021): 002181, 10.1136/jitc-2020-002181. PMC8183210 34083417

[217] A. H. Lee , L. Sun , A. Y. Mochizuki , et al., “Neoadjuvant PD‐1 Blockade Induces T Cell and cDC1 Activation but Fails to Overcome the Immunosuppressive Tumor Associated Macrophages in Recurrent Glioblastoma,” Nature Communications 12, no. 1 (2021): 6938, 10.1038/s41467-021-26940-2. PMC8626557 34836966

[218] K. Woroniecka , P. Chongsathidkiet , K. Rhodin , et al., “T‐Cell Exhaustion Signatures Vary With Tumor Type and Are Severe in Glioblastoma,” Clinical Cancer Research 24, no. 17 (2018): 4175–4186, 10.1158/1078-0432.CCR-17-1846. PMC6081269 29437767

[219] S. Tu , X. Lin , J. Qiu , et al., “Crosstalk between Tumor‐Associated Microglia/Macrophages and CD8‐Positive T Cells Plays a Key Role in Glioblastoma,” Frontiers in Immunology 12 (2021): 650105, 10.3389/fimmu.2021.650105. PMC8358794 34394072

[220] E. C. F. Yeo , M. P. Brown , T. Gargett , and L. M. Ebert , “The Role of Cytokines and Chemokines in Shaping the Immune Microenvironment of Glioblastoma: Implications for Immunotherapy,” Cells 10, no. 3 (2021): 607, 10.3390/cells10030607. PMC8001644 33803414

[221] D. A. Wainwright , S. Sengupta , Y. Han , and M. S. Lesniak , “Thymus‐derived Rather Than Tumor‐induced Regulatory T Cells Predominate in Brain Tumors,” Neuro‐Oncology 13, no. 12 (2011):1308–1323, https://academic.oup.com/neuro‐oncology/article/13/12/1308/1084694. 10.1093/neuonc/nor134 PMC3223094 21908444

[222] L. Zhai , E. Ladomersky , K. Lauing , et al., “Infiltrating T Cells Increase IDO1 Expression in Glioblastoma and Contribute to Decreased Patient Survival,” Clinical Cancer Research 23, no. 21 (2017): 6650–6660, https://aacrjournals.org/clincancerres/article/23/21/6650/260838/Infiltrating‐T‐Cells‐Increase‐IDO1‐Expression‐in. 10.1158/1078-0432.CCR-17-0120 PMC5850948 28751450

[223] Y. Jia , L. Liu , and B. Shan , “Future of Immune Checkpoint Inhibitors: Focus on Tumor Immune Microenvironment,” Annals of Translational Medicine 8, no. 17 (2020): 1095, 10.21037/atm-20-3735. PMC7575936 33145314

[224] J. Lohr , T. Ratliff , A. Huppertz , et al., “Effector T‐Cell Infiltration Positively Impacts Survival of Glioblastoma Patients and Is Impaired by Tumor‐Derived TGF‐β,” Clinical Cancer Research 17, no. 13 (2011): 4296–4308, https://aacrjournals.org/clincancerres/article/17/13/4296/11967/Effector‐T‐Cell‐Infiltration‐Positively‐Impacts. 10.1158/1078-0432.CCR-10-2557 21478334

[225] S. Valdebenito , S. Malik , R. Luu , et al., “Tunneling Nanotubes, TNT, Communicate Glioblastoma With Surrounding Non‐tumor Astrocytes to Adapt Them to Hypoxic and Metabolic Tumor Conditions,” Scientific Reports 11, no. 1 (2021): 14556, 10.1038/s41598-021-93775-8. PMC8282675 34267246

[226] L. Simone , D. L. Capobianco , F. Di Palma , et al., “GFAP Serves as a Structural Element of Tunneling Nanotubes Between Glioblastoma Cells and Could Play a Role in the Intercellular Transfer of Mitochondria,” Frontiers in Cell and Developmental Biology 11 (2023): 1221671, 10.3389/fcell.2023.1221671. PMC10598779 37886397

[227] S. McCutcheon and D. C. Spray , “Glioblastoma–Astrocyte Connexin 43 Gap Junctions Promote Tumor Invasion,” Molecular Cancer Research 20, no. 2 (2022): 319–331, https://aacrjournals.org/mcr/article/20/2/319/678055/Glioblastoma‐Astrocyte‐Connexin‐43‐Gap‐Junctions. 10.1158/1541-7786.MCR-21-0199 PMC8816813 34654721

[228] M. Osswald , E. Jung , F. Sahm , et al., “Brain Tumour Cells Interconnect to a Functional and Resistant Network,” Nature 528 (2015): 93–98, https://www.nature.com/articles/nature16071. 10.1038/nature16071 26536111

[229] D. Truong , R. Fiorelli , E. S. Barrientos , et al., “A Three‐dimensional (3D) Organotypic Microfluidic Model for Glioma Stem Cells—Vascular Interactions,” Biomaterials 198 (2019): 63–77, 10.1016/j.biomaterials.2018.07.048. PMC6353712 30098794

[230] X. Fan , K. Hou , G. Liu , R. Shi , W. Wang , and G. Liang , “Strategies to Overcome the Limitations of Current Organoid Technology—engineered Organoids,” Journal of Tissue Engineering 16 (2025): 20417314251319475, 10.1177/20417314251319475. PMC12033597 40290859

[231] Y. Chonan , S. Taki , O. Sampetrean , H. Saya , and R. Sudo , “Endothelium‐Induced Three‐Dimensional Invasion of Heterogeneous Glioma Initiating Cells in a Microfluidic Coculture Platform,” Integrative Biology 9, no. 9 (2017): 762–773, 10.1039/c7ib00091j. 28752870

[232] P. G. Gritsenko and P. Friedl , “Adaptive Adhesion Systems Mediate Glioma Cell Invasion in Complex Environments,” Journal of Cell Science 131, no. 15 (2018): jcs216382, 10.1242/jcs.216382. PMC6104823 29991514

[233] B. Cakir , Y. Xiang , Y. Tanaka , et al., “Engineering of human Brain Organoids With a Functional Vascular‐Like System,” Nature Methods 16 (2019): 1169–1175, https://www.nature.com/articles/s41592‐019‐0586‐5. 10.1038/s41592-019-0586-5 PMC6918722 31591580

[234] J. F. Navarro , S. Crilly , W. K. Chan , et al., “Cerebral Organoids With Integrated Endothelial Networks Emulate the Neurovascular Unit and Mitigate Core Necrosis,” Advanced Science 12 (2025): 07256, https://advanced.onlinelibrary.wiley.com/doi/10.1002/advs.202507256. 10.1002/advs.202507256 PMC12631921 40884248

[235] A. Nakhlband , “Organoid‐Engineered Neurovascular Units for Drug Discovery and Neurodegeneration Research,” Bioimpacts 15 (2025): 33140, 10.34172/bi.33140. PMC12913201 41716517

[236] Y. Shi , L. Sun , M. Wang , et al., “Vascularized human Cortical Organoids (vOrganoids) Model Cortical Development in Vivo,” PLoS Biology 18, no. 5 (2020): 3000705, 10.1371/journal.pbio.3000705. PMC7250475 32401820

[237] M. Ishahak , R. H. Han , D. Annamalai , et al., “Genetically Engineered Brain Organoids Recapitulate Spatial and Developmental States of Glioblastoma Progression,” Advanced Science 12, no. 10 (2025): 2410110, 10.1002/advs.202410110. PMC11905097 39836549

[238] C. Wang , N. Stöffler , and H.‐K. Liu , “Laboratory‐Engineered Glioblastoma Organoid Culture and Drug Screening,” Journal of Visualized Experiments (JoVE), no. 215 (2025): 67593, 10.3791/67593. 39868688

[239] Y.‐T. Tung , Y.‐C. Chen , K. Derr , et al., “A 3D Bioprinted Human Neurovascular Unit Model of Glioblastoma Tumor Growth,” Advanced Healthcare Materials 13 (2024): 2302831, https://advanced.onlinelibrary.wiley.com/doi/10.1002/adhm.202302831. 10.1002/adhm.202302831 PMC11176035 38394389

[240] E. Maloney , C. Clark , H. Sivakumar , et al., “Immersion Bioprinting of Tumor Organoids in Multi‐Well Plates for Increasing Chemotherapy Screening Throughput,” Micromachines (Basel) 11, no. 2 (2020): 208, https://www.mdpi.com/2072‐666X/11/2/208. 10.3390/mi11020208 PMC7074680 32085455

[241] M. Tang , S. Jiang , X. Huang , et al., “Integration of 3D Bioprinting and Multi‐algorithm Machine Learning Identified Glioma Susceptibilities and Microenvironment Characteristics,” Cell Discovery 10, no. 1 (2024): 39, 10.1038/s41421-024-00650-7. PMC11003988 38594259

[242] I. K. Koh , J. Cha , J. Park , J. Choi , S.‐G. Kang , and P. Kim , “The Mode and Dynamics of Glioblastoma Cell Invasion Into a Decellularized Tissue‐derived Extracellular Matrix‐based Three‐dimensional Tumor Model,” Scientific Reports 8 (2018): 4608, https://www.nature.com/articles/s41598‐018‐22681‐3. 10.1038/s41598-018-22681-3 PMC5854588 29545552

[243] C. Wang , Q. Zhao , X. Zheng , et al., “Decellularized Brain Extracellular Matrix Slice Glioblastoma Culture Model Recapitulates the Interaction Between Cells and the Extracellular Matrix Without a Nutrient‐oxygen Gradient Interference,” Acta Biomaterialia 158 (2023): 132–150, 10.1016/j.actbio.2022.12.044. 36565784

[244] Fondazione Policlinico Universitario Agostino Gemelli IRCCS , “Development and Characterization of Patient's Derived Organoids as a Platform for the Screening of Novel Therapeutic Treatments for Glioblastoma Multiforme,” clinicaltrials.gov, Clinical trial registration NCT06781372, 2025. Accessed: May 04, 2026. [Online]. Available: https://clinicaltrials.gov/study/NCT06781372.

[245] K. H. Kim , “Patient‐derived Organoids as Predictive Models for Drug Response Testing and Repurposing in Glioblastoma Therapy,” clinicaltrials.Gov, Clinical Trial Registration NCT06782984, 2025. Accessed: May 04, 2026. [Online]. Available: https://clinicaltrials.gov/study/NCT06782984.

[246] Maastricht Radiation Oncology , “Glioma Stem Cell Organoids: Preclinical Model of Glioblastoma Heterogeneity to Explore Resistance Mechanisms to Conventional Treatment Schedules.,” clinicaltrials.Gov, Clinical Trial Registration NCT04868396, 2024. Accessed: May 04, 2026. [Online]. Available: https://clinicaltrials.gov/study/NCT04868396.

[247] C. H. Becquerel , “Feasibility of ORGAnoids in Routine Clinical Practice for Molecular Analysis of the GLIOvascular Niche in Patients with Primary Brain Tumors.,” clinicaltrials.Gov, Clinical Trial Registration NCT07029100, 2025. Accessed: May 04, 2026. [Online]. Available: https://clinicaltrials.gov/study/NCT07029100.

[248] Fondazione Policlinico Universitario Agostino Gemelli IRCCS , “Grafts of Patient‐Derived Glioblastoma Stem Cells onto Autologous Brain Organoids. A Precision Medicine Model for Testing Drugs against Tumor Invasion,” clinicaltrials.Gov, Clinical Trial Registration NCT05772741, 2023. Accessed: May 04, 2026. [Online]. Available: https://clinicaltrials.gov/study/NCT05772741.

[249] S. Baisiwala , E. Fazzari , M. X. Li , et al., “A human Tumor‐immune Organoid Model of Glioblastoma,” Cell Reports 45, no. 1 (2026): 116790, 10.1016/j.celrep.2025.116790. PMC12969483 41505256

[250] M. Khabibov , A. Garifullin , Y. Boumber , et al., “Signaling Pathways and Therapeutic Approaches in Glioblastoma Multiforme (Review),” International Journal of Oncology 60, no. 6 (2022): 1–18, 10.3892/ijo.2022.5359. PMC9084550 35445737

[251] O. Shafi and G. Siddiqui , “Tracing the Origins of Glioblastoma by Investigating the Role of Gliogenic and Related Neurogenic Genes/Signaling Pathways in GBM Development: A Systematic Review,” World Journal of Surgical Oncology 20, no. 1 (2022): 146, 10.1186/s12957-022-02602-5. PMC9087910 35538578

[252] S. Osuka , D. Zhu , Z. Zhang , et al., “JCI—N‐cadherin Upregulation Mediates Adaptive Radioresistance in Glioblastoma,” The Journal of Clinical Investigation 131 (2021): 136098, https://www.jci.org/articles/view/136098. 10.1172/JCI136098 PMC7954595 33720050

[253] T.‐J. Kao , C.‐L. Lin , W.‐B. Yang , H.‐Y. Li , and T.‐I. Hsu , “Dysregulated Lipid Metabolism in TMZ‐resistant Glioblastoma: Pathways, Proteins, Metabolites and Therapeutic Opportunities,” Lipids in Health and Disease 22 (2023): 114, https://link.springer.com/article/10.1186/s12944‐023‐01881‐5. 10.1186/s12944-023-01881-5 PMC10398973 37537607

[254] C. Lu , T. Kang , J. Zhang , et al., “Combined Targeting of Glioblastoma Stem Cells of Different Cellular States Disrupts Malignant Progression,” Nature Communications 16 (2025): 2974, https://www.nature.com/articles/s41467‐025‐58366‐5. 10.1038/s41467-025-58366-5 PMC11947120 40140646

[255] H.‐G. Yi , Y. H. Jeong , Y. Kim , et al., “A Bioprinted Human‐Glioblastoma‐on‐a‐chip for the Identification of Patient‐Specific Responses to Chemoradiotherapy,” Nature Biomedical Engineering 3 (2029): 509–519, https://www.nature.com/articles/s41551‐019‐0363‐x. 10.1038/s41551-019-0363-x 31148598

[256] A. Bentaberry‐Rosa , Y. Nicaise , C. Delmas , et al., “Overexpression of Growth Differentiation Factor 15 in Glioblastoma Stem Cells Promotes Their Radioresistance,” Cancers 16, no. 1 (2024): 27, 10.3390/cancers16010027. PMC10778311 38201456

[257] M. J. Rybin , M. E. Ivan , N. G. Ayad , and Z. Zeier , “Organoid Models of Glioblastoma and Their Role in Drug Discovery,” Frontiers in Cellular Neuroscience 15 (2021): 605255, 10.3389/fncel.2021.605255. PMC7892608 33613198

[258] R. Gómez‐Oliva , S. Domínguez‐García , L. Carrascal , L. Carrascal , et al., “Evolution of Experimental Models in the Study of Glioblastoma: Toward Finding Efficient Treatments,” Frontiers in oncology 10 (2021): 614295, 10.3389/fonc.2020.614295. PMC7878535 33585240

[259] I. Pediaditakis , K. R. Kodella , D. V. Manatakis , et al., “A Microengineered Brain‐Chip to Model Neuroinflammation in Humans,” iScience 25, no. 8 (2022): 104813, 10.1016/j.isci.2022.104813. PMC9379671 35982785

[260] S. Chen , L. Wang , L. Yang , A. S. Rana , and C. He , “Engineering Biomimetic Microenvironment for Organoid,” Macromolecular Bioscience 23, no. 12 (2023): 2300223, 10.1002/mabi.202300223. 37531622

[261] M. Verduin , L. Hoosemans , M. Vanmechelen , et al., “Patient‐derived Glioblastoma Organoids Reflect Tumor Heterogeneity and Treatment Sensitivity,” Neuro‐Oncology Advances 5, no. 1 (2023): vdad152, 10.1093/noajnl/vdad152. PMC10733660 38130902

[262] N. S. Bayin , J. D. Frenster , R. Sen , et al., “Notch Signaling Regulates Metabolic Heterogeneity in Glioblastoma Stem Cells,” Oncotarget 8, no. 39 (2017): 64932–64953, 10.18632/oncotarget.18117. PMC5630302 29029402

[263] C. Xu , X. Yuan , P. Hou , et al., “Development of Glioblastoma Organoids and Their Applications in Personalized Therapy,” Cancer Biology & Medicine 20, no. 5 (2023): 353–368, 10.20892/j.issn.2095-3941.2023.0061. PMC10246443 37283493

[264] B. Mann , N. Artz , R. Darawsheh , D. E. Kram , S. Hingtgen , and A. B. Satterlee , “Opportunities and Challenges for Patient‐Derived Models of Brain Tumors in Functional Precision Medicine,” npj Precision Oncology 9 (2025): 47, https://www.nature.com/articles/s41698‐025‐00832‐w. 10.1038/s41698-025-00832-w PMC11828933 39953052

[265] G. A. Gomez , M. Oksdath , M. P. Brown , and L. M. Ebert , “New Approaches to Model Glioblastoma in Vitro Using Brain Organoids: Implications for Precision Oncology,” Translational Cancer Research 8, no. 6 (2019): S606–S611, 10.21037/tcr.2019.09.08. PMC8798484 35117142

[266] J. A. Benitez , D. Finlay , A. Castanza , et al., “PTEN Deficiency Leads to Proteasome Addiction: A Novel Vulnerability in Glioblastoma,” Neuro‐Oncology 23, no. 7 (2021): 1072–1086, 10.1093/neuonc/noab001. PMC8661409 33428749

[267] M.‐D. Papaioannou , K. Sangster , R. S. Sajid , U. Djuric , and P. Diamandis , “Cerebral Organoids: Emerging Ex Vivo Humanoid Models of Glioblastoma,” Acta Neuropathologica Communications 8 (2020): 209, 10.1186/s40478-020-01077-3. PMC7706050 33261657

[268] J. Kopinja , R. S. Sevilla , D. Levitan , et al., “A Brain Penetrant Mutant IDH1 Inhibitor Provides In Vivo Survival Benefit,” Scientific Reports 7 (2017): 13853, https://www.nature.com/articles/s41598‐017‐14065‐w. 10.1038/s41598-017-14065-w PMC5653818 29062039

[269] G. Cutshaw , S. Uthaman , N. Hassan , S. Kothadiya , X. Wen , and R. Bardhan , “The Emerging Role of Raman Spectroscopy as an Omics Approach for Metabolic Profiling and Biomarker Detection Toward Precision Medicine,” Chemical Reviews 123, no. 13 (2023): 8297–8346, 10.1021/acs.chemrev.2c00897. PMC10626597 37318957

[270] M. Riva , T. Sciortino , R. Secoli , et al., “Glioma Biopsies Classification Using Raman Spectroscopy and Machine Learning Models on Fresh Tissue Samples,” Cancers 13, no. 5 (2021): 1073, 10.3390/cancers13051073. PMC7959285 33802369

[271] S. Maramraju , A. Kowalczewski , A. Kaza , et al., “AI‐Organoid Integrated Systems for Biomedical Studies and Applications,” Bioengineering & Translational Medicine 9, no. 2 (2024): 10641, 10.1002/btm2.10641. PMC10905559 38435826

[272] H. Shi , A. Kowalczewski , D. Vu , et al., “Organoid Intelligence: Integration of Organoid Technology and Artificial Intelligence in the New Era of in Vitro Models,” Medicine in Novel Technology and Devices 21 (2024): 100276, 10.1016/j.medntd.2023.100276. PMC11027187 38646471

[273] A. Jose , P. Kulkarni , J. Thilakan , et al., “Integration of Pan‐omics Technologies and Three‐dimensional in Vitro Tumor Models: An Approach Toward Drug Discovery and Precision Medicine,” Molecular Cancer 23 (2024): 50, https://link.springer.com/article/10.1186/s12943‐023‐01916‐6. 10.1186/s12943-023-01916-6 PMC10924370 38461268

[274] H. Zhou , B. Chen , L. Zhang , and C. Li , “Machine Learning‐based Identification of Lower Grade Glioma Stemness Subtypes Discriminates Patient Prognosis and Drug Response,” Computational and Structural Biotechnology Journal 21 (2023): 3827–3840, 10.1016/j.csbj.2023.07.029. PMC10407594 37560125

[275] X. Xie , X. Li , and W. Song , “Tumor Organoid Biobank‐new Platform for Medical Research,” Scientific Reports 13, no. 1 (2023): 1819, 10.1038/s41598-023-29065-2. PMC9892604 36725963

[276] S. Qu , R. Xu , G. Yi , et al., “Patient‐derived Organoids in human Cancer: A Platform for Fundamental Research and Precision Medicine,” Molecular Biomedicine 5, no. 1 (2024): 6, 10.1186/s43556-023-00165-9. PMC10859360 38342791

[277] K. Tubbesing , N. Moskwa , T. C. Khoo , et al., “Raman Microspectroscopy Fingerprinting of Organoid Differentiation state,” Cellular & Molecular Biology Letters 27, no. 1 (2022): 53, 10.1186/s11658-022-00347-3. PMC9238268 35764935

[278] K. Ember , F. Dallaire , A. Plante , et al., “In Situ Brain Tumor Detection Using a Raman Spectroscopy System—Results of a Multicenter Study,” Scientific Reports 14 (2024): 13309, https://www.nature.com/articles/s41598‐024‐62543‐9. 10.1038/s41598-024-62543-9 PMC11164901 38858389

[279] E. V. Stupak , V. M. Glotov , A. S. Askandaryan , et al., “Raman Spectroscopy in the Diagnosis of Brain Gliomas: A Literature Review,” Cureus 17, no. 2: 79165, 10.7759/cureus.79165. PMC11921993 40109807

[280] X. Du , Z. Chen , Q. Li , et al., “Organoids Revealed: Morphological Analysis of the Profound next Generation in‐vitro Model With Artificial Intelligence,” Bio‐Design and Manufacturing 6, no. 3 (2023): 319–339, 10.1007/s42242-022-00226-y. PMC9867835 36713614

[281] X. Bian , G. Li , C. Wang , et al., “A Deep Learning Model for Detection and Tracking in High‐throughput Images of Organoid,” Computers in Biology and Medicine 134 (2021): 104490, 10.1016/j.compbiomed.2021.104490. 34102401

[282] S. Migliozzi , Y. T. Oh , M. Hasanain , et al., “Integrative Multi‐omics Networks Identify PKCδ and DNA‐PK as Master Kinases of Glioblastoma Subtypes and Guide Targeted Cancer Therapy,” Nature Cancer 4, no. 2 (2023): 181–202, 10.1038/s43018-022-00510-x. PMC9970878 36732634

[283] G. Mestrallet , “Predicting Immunotherapy Outcomes in Glioblastoma Patients Through Machine Learning,” Cancers 16, no. 2 (2024): 408, 10.3390/cancers16020408. PMC10813889 38254897

[284] E. Zihni , V. I. Madai , M. Livne , et al., “Opening the Black Box of Artificial Intelligence for Clinical Decision Support: A Study Predicting Stroke Outcome,” PLoS ONE 15, no. 4 (2020): 0231166, 10.1371/journal.pone.0231166. PMC7135268 32251471

[285] S. Khalighi , K. Reddy , A. Midya , K. B. Pandav , A. Madabhushi , and M. Abedalthagafi , “Artificial Intelligence in Neuro‐Oncology: Advances and Challenges in Brain Tumor Diagnosis, Prognosis, and Precision Treatment,” npj Precision Oncology 8, no. 1 (2024): 80, 10.1038/s41698-024-00575-0. PMC10980741 38553633

[286] S. Bakas , D. R. Ormond , K. D. Alfaro‐Munoz , et al., “iGLASS: Imaging Integration Into the Glioma Longitudinal Analysis Consortium,” Neuro‐Oncology 22, no. 10 (2020): 1545–1546, 10.1093/neuonc/noaa160. PMC7566469 32644158

[287] F. P. Barthel , K. C. Johnson , F. S. Varn , et al., “Longitudinal Molecular Trajectories of Diffuse Glioma in Adults,” Nature 576 (2019): 112–120, https://www.nature.com/articles/s41586‐019‐1775‐1. 10.1038/s41586-019-1775-1 PMC6897368 31748746

[288] H. H.‐F. Loong , A. M. Wong , D. T.‐M. Chan , et al., “Patient‐derived Tumor Organoid Predicts Drugs Response in Glioblastoma: A Step Forward in Personalized Cancer Therapy?,” Journal of Clinical Neuroscience 78 (2020): 400–402, 10.1016/j.jocn.2020.04.107. 32340843

[289] C.‐C. Chen , H.‐W. Li , Y.‐L. Wang , et al., “Patient‐derived Tumor Organoids as a Platform of Precision Treatment for Malignant Brain Tumors,” Scientific Reports 12, no. 1 (2022): 16399, 10.1038/s41598-022-20487-y. PMC9525286 36180511

[290] R. H. Utama , L. Atapattu , A. P. O'Mahony , et al., “A 3D Bioprinter Specifically Designed for the High‐Throughput Production of Matrix‐Embedded Multicellular Spheroids,” iScience 23, no. 10 (2020): 101621, 10.1016/j.isci.2020.101621. PMC7567958 33089109

[291] H. Renner , M. Grabos , K. J. Becker , et al., “A Fully Automated High‐Throughput Workflow for 3D‐based Chemical Screening in human Midbrain Organoids,” eLife 9 (2020): 52904, 10.7554/eLife.52904. PMC7609049 33138918

[292] P. J. Tebon , B. Wang , A. L. Markowitz , et al., “Drug Screening at Single‐organoid Resolution via Bioprinting and Interferometry,” Nature Communications 14, no. 1 (2023): 3168, 10.1038/s41467-023-38832-8. PMC10244450 37280220

[293] K. Zhao , P. Calero‐Pérez , M. H. A. Bopp , et al., “Correlation of MR‐Based Metabolomics and Molecular Profiling in the Tumor Microenvironment of Temozolomide‐Treated Orthotopic GL261 Glioblastoma in Mice,” International Journal of Molecular Sciences 24, no. 24 (2023): 17628, 10.3390/ijms242417628. PMC10743933 38139457

[294] A. P. Sahu , K. Palsa , G. Shenoy , et al., “Immu‐06. H‐Ferritin Affects Immune Cell Infiltration into the Glioblastoma Tumor Microenvironment in a Sex‐Biased Manner,” Neuro‐Oncology 26, no. 8 (2024): viii152–viii153, 10.1093/neuonc/noae165.0599.

[295] R. Raman , V. Prabhu , P. Kumar , and N. K. Mani , “Advancements in Microfluidic Platforms for Glioblastoma Research,” Chemistry 6, no. 5 (2024): 1039–1062, 10.3390/chemistry6050060.

[296] A. P. Sahu , K. Palsa , G. Shenoy , B. Slagle‐Webb , and J. R. Connor , “Tmet‐09. SEX‐Biased Role of Tumor‐Associated Macrophages in Glioblastoma Iron Metabolism,” Neuro‐Oncology 26, no. 8 (2024): viii289, 10.1093/neuonc/noae165.1147. PMC13076414 41973350

[297] A. P. Sahu , K. Palsa , G. Shenoy , B. Slagle‐Webb , and J. R. Connor , “Tumor‐associated Macrophages Protect Glioblastoma Cells From Ferroptosis by Inducing the Release of Ferritin‐bound Iron via Exosomes,” BioRxiv (2025): 683804, 10.1101/2025.10.21.683804.

